# Cancer-initiating cells derived from established cervical cell lines exhibit stem-cell markers and increased radioresistance

**DOI:** 10.1186/1471-2407-12-48

**Published:** 2012-01-28

**Authors:** Jacqueline López, Adela Poitevin, Veverly Mendoza-Martínez, Carlos Pérez-Plasencia, Alejandro García-Carrancá

**Affiliations:** 1 Programa de Doctorado en Ciencias Bioquímicas, Facultad de Química, Universidad Nacional Autónoma de México, Mexico City, Mexico; 2 Hospital Médica Sur, Unidad de Radioterapia, Mexico City, Mexico; 3 Laboratorio de Oncología Genómica, Instituto Nacional de Cancerología & UBIMED-FESI, Universidad Nacional Autónoma de México, Mexico City, Mexico; 4 Unidad de Investigación Biomédica en Cáncer, División de Investigación Básica, Instituto Nacional de Cancerología, Secretaría de Salud & Instituto de Investigaciones Biomédicas, Universidad Nacional Autónoma de México, Mexico City, Mexico

**Keywords:** Cancer-initiating cells, Cervical cancer, Stem cell markers, Radioresistance, Epithelia to mesenchymal transition

## Abstract

**Background:**

Cancer-initiating cells (CICs) are proposed to be responsible for the generation of metastasis and resistance to therapy. Accumulating evidences indicates CICs are found among different human cancers and cell lines derived from them. Few studies address the characteristics of CICs in cervical cancer. We identify biological features of CICs from four of the best-know human cell lines from uterine cervix tumors. (HeLa, SiHa, Ca Ski, C-4 I).

**Methods:**

Cells were cultured as spheres under stem-cell conditions. Flow cytometry was used to detect expression of CD34, CD49f and CD133 antigens and Hoechst 33342 staining to identify side population (SP). Magnetic and fluorescence-activated cell sorting was applied to enrich and purify populations used to evaluate tumorigenicity in nude mice. cDNA microarray analysis and *in vitro *radioresistance assay were carried out under standard conditions.

**Results:**

CICs, enriched as spheroids, were capable to generate reproducible tumor phenotypes in nu-nu mice and serial propagation. Injection of 1 × 10^3 ^dissociated spheroid cells induced tumors in the majority of animals, whereas injection of 1 × 10^5 ^monolayer cells remained nontumorigenic. Sphere-derived CICs expressed CD49f surface marker. Gene profiling analysis of HeLa and SiHa spheroid cells showed up-regulation of CICs markers characteristic of the female reproductive system. Importantly, epithelial to mesenchymal (EMT) transition-associated markers were found highly expressed in spheroid cells. More importantly, gene expression analysis indicated that genes required for radioresistance were also up-regulated, including components of the double-strand break (DSB) DNA repair machinery and the metabolism of reactive oxygen species (ROS). Dose-dependent radiation assay indicated indeed that CICs-enriched populations exhibit an increased resistance to ionizing radiation (IR).

**Conclusions:**

We characterized a self-renewing subpopulation of CICs found among four well known human cancer-derived cell lines (HeLa, SiHa, Ca Ski and C-4 I) and found that they express characteristic markers of stem cell, EMT and radioresistance. The fact that CICs demonstrated a higher degree of resistance to radiation than differentiated cells suggests that specific detection and targeting of CICs could be highly valuable for the therapy of tumors from the uterine cervix.

## Background

On the global scale, cervical cancer represents the second most frequent cancer in women with approximately 530,000 new cases registered each year and >274,000 deaths worldwide [1].

Currently, approximately 35% of women diagnosed with cervical cancer have recurrent disease, with 90% of these found within 3 years after the initial treatment [2]. Thus, improved targeted therapies and chemo-radiosensitization strategies are essential for reducing the mortality of this devastating malignancy.

One emerging model for the development of drug and radio-resistant invokes the existence within tumors of a pool of self-renewing malignant progenitors known as cancer-initiating cells (CICs), at the top of a hierarchy from which cells with distinct degrees of differentiation derive [3]. If CICs are more resistant to radiation, relapse after partial remission could likely be due to failure to eradicate these cells which, despite bulk tumor shrinkage can subsequently reproduce the entire malignant phenotype [4]. Indeed, normal stem cells have been shown to possess several characteristics that confer chemo- and radioresistance [4,5].

CICs were first identified in acute myeloid leukemia as possessing the cell surface antigenic phenotype CD34^+^CD38‒ and the capacity to reproduce the complete leukemic hierarchy upon xenoengraftment [6,7]. Similar to the hematopoietic system, epithelial linings of the majority of tissue surfaces undergo continuous turnover and are organized according to a stem cell hierarchy [3]. In 2003, CD24‒CD44^+ ^cells were isolated from human breast tumors that could be serially propagated in animals and could recapitulate their original phenotype [8]. CICs have since been identified from numerous other epithelial malignancies [9-12]. A consensus of five defining criteria has been established to affirm the existence of CICs: (a) capacity to self-renew; (b) tumorigenic potential to recreate the full phenotypic heterogeneity of the parent tumor; (c) expression of distinctive cells markers; (d) differentiation into nontumorigenic cells, and (e) restriction to a small minority of the total tumor population [3,13]. However, concerning this last feature the use of highly immunocompromised NOD/SCID interleukin-2 receptor gamma chain null mice, has shown that the detection of tumorigenic melanoma cells can be increased by several orders of magnitude [14].

In the case of cervical cancer, Feng et al. [15], isolated 8/19 tumor-derived cultures encompassing stem-like cells capable of self-renewal and extensive proliferation as clonal nonadherent spherical clusters. Xenoengraftment of 1 × 10^5 ^dissociated spheroid cells allowed full recapitulation of the original tumor, whereas the same amount of non-selected tumor cells remained non-tumorigenic remained non-tumorigenic. Furthermore, Sox2 staining was detected in the majority of tumor sphere cells isolated from fresh cervical cancer tissues but not from the differentiated cells. When Sox2 was stably expressed in cervical cancer cells (SiHa and HeLa), overexpressing cells had increased proliferation, clonogenicity, and tumorigenicity *in vitro *and *in vivo *[16].

Established cancer cell lines can be maintained indefinitely in culture and usually can form tumors when transplanted *in vivo*. Cancer cell lines are attractive and reproducible models for cancer studies and represent an alternative source for CICs research. However, studies with well -established cervical cell lines that allow a better understanding of CICs are still needed. The objective of this study was to isolate and characterize cervical CICs (CCICs) from four well-known established cell lines. These cells are fully capable of self renewing and of serially propagating tumors in nu-nu mice. CCICs-enriched spheroids fulfill currently accepted criteria for the existence of a subpopulation of CICs [3,13]. Gene profiling analysis showed increased expression of adult CICs markers, genes involved in DNA double-strand breaks (DSB) repair and genes involved in an epithelial to mesenchymal transition. Importantly, sphere-forming CCICs demonstrated a higher degree of radioresistance than differentiated cells suggesting that specific detection and targeting of CICs could be highly valuable for the therapy of recurrent chemo-radioresistant tumors of the uterine cervix.

## Methods

### Cell culture

HeLa, SiHa, Ca Ski, and C-4 I human cervical cell lines were maintained in Dubelcco's modified Eagle's medium (DMEM, Gibco) supplemented with 10% fetal bovine serum (FBS, Gibco), 100 units/ml penicillin (Gibco) and 100 μg/ml streptomycin (Gibco). In all experiments, cells were maintained at 37°C, 5% CO_2_, and 95% air atmosphere. All of our experiments were performed on cultures that were 70% confluent.

### Culture and passage of tumor spheres

Cells were placed in serum-free medium (SFM) mixed with 20 ng/ml epidermal growth factor (EGF, Invitrogen), 20 ng/ml basic fibroblast growth factor (bFGF, Invitrogen), and 0.4% bovine serum albumin (BSA, Sigma-Aldrich) at a density of 1 × 10^3 ^cells/ml and cultured in ultra low attachment plates (Costar). The sphere culture media was changed every 48 h until the majority of spheres reached 100-400 μm in diameter (approximately 7 days). Spheres were collected by gentle centrifugation (5 min at 2,500 rpm), dissociated with EDTA 1.0 mM, and mechanically disrupted with a fire-polished Pasteur pipette. The cell suspension was sieved through a cell strainer with 40-μm nylon mesh to achieve a single-cell suspension and then re-plated in complete fresh medium.

### Sphere-forming efficiency assay

For analysis of sphere formation, subconfluent cells were thoroughly dissociated with EDTA to prepare a single-cell suspension that was always visually verified. Then, 100 cells per well were plated in 96-well culture dishes in 200 μl of growth medium, and 25 μl of medium per well was added every 2 days. The number of spheres for each well was evaluated 7 days after seeding and sphere formation rate was counted. Sphere-forming efficiency (SFE) was calculated as the number of spheres formed divided by the original number of single cells seeded and expressed as a percentage [17]. SFE was calculated from first through the fifth generation. All experiments were done in triplicate.

### Differentiation assay

To examine cervical tumor-like epithelial differentiation of anchorage-independent cells, spheres were dissociated with EDTA and single cells were plated onto glass cover slips pre-coated with poly-L-lysine (Sigma-Aldrich) under standard conditions, DMEM/F12 supplemented with 10% FBS without growth factors. Cells were placed on cover slips, fed with FBS-supplemented medium every 2 days and processed 7 days after plating. Immunocytochemistry was performed with human anti-cytokeratin (AE1/3 clone, 1:50 final dilution, Dako) and visualized with LSAB + System-HRP (Dako). The chromogene was 3,3'-diaminobenzidine tetrahydrochlorate (Dako) solution developed with H_2_O_2_. Sections were counterstained with hematoxylin, dehydrated and cleared in xylene, and then mounted with Eukitt medium.

### Clonogenic assay

Each well (35-mm) of a six-well culture dish was coated with 2 ml bottom agar-medium mixture (DMEM, 10% FBS, 0.6% agar). After solidifying, 2 ml top agar-medium mixture (DMEM, 10% FBS, 0.3% Noble agar, BD Difco) containing cells was added, and dishes were incubated at 37°C for 21 days. Colonies were fixed with 6.0% glutaraldehyde, stained with 0.5% crystal violet, and counted using a dissecting microscope to determinate plating efficiency (PE).

### Surface marker analysis by flow cytometry

One million cells were incubated for 10 min at 4°C with FcR blocking reagent (Miltenyi Biotech), followed by labeling with monoclonal antibodies to CD34 (AC136 clone, 1:11 dilution, Miltenyi Biotec), CD49f (GoH3 clone, 1:11 dilution, Becton Dickinson) and CD133 (AC133/1 clone, 1:11 dilution, Miltenyi Biotec). After 15 min at 4°C, cells were extensively washed with ice-cold PBS and resuspended in 0.5 μg/ml propidium iodide (PI) for 10 min at room temperature, and shielded from light prior to flow cytometry analysis with FACSCalibur (BD Biosciences, CA, USA) and Cell Quest Pro software (BD Biosciences, CA, USA). Fluorochrome-conjugated isotype-matched monoclonal antibodies from the same manufacturers were utilized to establish background fluorescence. CD133^+ ^and CD34^+ ^hematopoietic stem cells from umbilical cord blood were obtained from consenting mothers after full-term delivery and used as positive controls for CD133 and CD34 labeling. Human peripheral blood leukocytes were used as positive control for CD49f labeling.

### Characterization of SP population

One millions of cells were resuspended in 1.0 ml of pre-warmed DMEM at 37°C containing 2% FBS, 1 mM Hepes, and 5 μg/ml Hoechst 33342 (Sigma-Aldrich) and were incubated under constant and slow agitation for 90 mn at 37°C. When Verapamil was used to block Hoechst efflux, cells were stained as described in the presence of 50 μM Verapamil (Sigma-Aldrich) [18]. After staining, cells were washed with Hank's buffered salt solution, resuspended in ice-cold staining media, and then maintained on ice until their analysis or sorting. Immediately before analysis, PI was added at a final concentration of 2 mg/ml. Bone marrow cells were used as positive control. Murine bone marrow was extracted from the femurs and tibias of C57B1/6 mice, a single-cell suspension was made by passage of the bone marrow through an 18-gauge needle, and the cells were pelleted by centrifugation. Analysis and sorting were performed with a MoFlo Cell Sorter (Beckman Coulter, CA, USA) employing 60-mW multiline ultraviolet (UV) from a coherent I-90 laser. Hoechst blue was detected at 424/24 nm and Hoechst red at 590/30. The channels were separated by a 555LP dichromic beam-splitter. Sorting was carried out using a 100-lm nozzle and at 35-W sheath pressure, utilizing the purity-1 mode. Dye effluxing cells appear as a low fluorescing population termed the side population (SP).

### Isolation by magnetic (MCS) and fluorescence cell sorting (FCS)

Human cervical cells lines were magnetically labeled and separated by double passage with 1 μl CD133 (CD133/1 clone, Miltenyi Biotec) and 1 μl CD34 microbeads (QBEND/10 clone, Miltenyi Biotec) per 1 × 10^6 ^cells, utilizing the Miltenyi Biotec CD34 and CD133 cell isolation kit. Indirect and positive separation was performed for CD49f using anti-phicoeritrin (PE) microbeads (PE4-14D10 clone, MiltenyiBiotec) and anti-CD49f-PE (GoH3 clone, Becton Dickinson). Magnetically enriched populations and SP were sorted with a MoFlo Cell Sorter (Beckman Coulter, CA, USA). At least 5,000 events were acquired for each sample and cells positive for PI were gated out. After separation by MACS and/or FACS, aliquots of positive and negative sorted populations were evaluated for purity by flow cytometry with a FACSCalibur (BD Biosciences, CA, USA) and Cell Quest software (BD Biosciences, CA, USA) using CD133/2-PE (239C3 clone, Miltenyi Biotec), CD49f (450-30A clone, Serotec), and CD34-PE antibodies (AC136 clone, Miltenyi Biotec). Purities ranged from 83 to 94% for positive and 89-99.7% for negative populations.

### In vivo xenograft experiments

All animal experiments adhered to the requirements of NOM-062-ZOO-1999 Mexican Official Law and protocols were approved by the Ethics Committee of the Biomedical Research Institute, UNAM. Dissociated cells obtained under adherent and nonadherent conditions were counted and resuspended in 100 μl of FBS and growth factor-free DMEM at different cell densities. Cells were injected subcutaneously (s.c.) into the left and right flanks of 4-6-week-old female nude athymic mice (BALB/c-nu/nu). Engrafted mice were inspected weekly for tumor appearance by visual observation and palpation. Six weeks after transplantation mice were euthanized and tumor tissue collected. Tumors were digested usually during 1 h using dispase solution (1 mg/ml, Invitrogen) and 0.5% collagenase Type I (Invitrogen). They were stirred slowly at 37°C until the tissue was completely dissolved. Dispersed cells were then separated from residual tissue by passing the mixture through a 40-μm cell strainer to produce a single-cell suspension. Cells were obtained by centrifugation and plated in SFM. After reformation and dissociation of spheroids, single cells were reinjected into mice and, similar to the tumor mincing studies, the entire process was repeated. The remaining tumor, fixed in 10% buffered formalin and embedded in paraffin was sectioned (5-μm) on a rotary microtome and subsequently stained with hematoxylin and eosin (H&E) for histological evaluation and immunohistochemistry. Immunostaining was performed using tissue sections mounted on poly-L-lysine-coated slides and dried at 37°C overnight. The slides were deparaffinized in xylene and rehydrated. Endogenous peroxidase was blocked with 3% H_2_O_2 _for 5 min. To reduce nonspecific binding, the sections were incubated with 20% normal goat serum for 30 min at room temperature. Cells expressing human epithelial cytokeratins were identified after overnight incubation at 4°C with anti-cytokeratin AE1/3, as described above.

### cDNA microarray analysis

Total RNA was extracted utilizing AllPrep DNA/RNA Micro Kit (Qiagen) and reverse-transcribed with the MessageAmp™ II aRNA Amplification Kit (Ambion) to generate Cy3-and Cy5-labeled (Ambion) cDNA probes for adherent and nonadherent samples, respectively. The labeled probes were hybridized to a cDNA Stanford University Microarray containing 40,000 immobilized oligonucleotide probes. Different fluorescently labeled cDNA probes were mixed in 45 μl of hybridization buffer DIG Easy Hyb (Roche), applied to the microarray and incubated at 37°C for 16 h. After hybridization slides were washed with 0.1 X SSC/0.1% SDS, 0.1 X SSC and 0.01 X SSC for 5 min at room temperature. Fluorescence intensities of Cy3 and Cy5 targets were measured and scanned separately using the GenePix 4100A Array Scanner (Axon Instruments, CA, USA). Images and quantitative data of gene-expression levels were processed and analyzed by GenePix Pro V5.0 (Axon Instruments, CA, USA). The array data for expression of 12,500 genes were filtered and genes with a median expression increased or decreased by a factor of at least 1.5-fold were selected. A total of 638 genes in HeLa and 857 genes in SiHa were clustered by the use of WebGestalt (Gene Set Analysis Toolkit). Two independent experiments were performed.

### Radioresistance assay

Cells from both monolayer cultures and 7-day-old spheres were enzymatically dissociated with EDTA and mechanically dissociated with a Pasteur pipette, both passed through a 40-μm sieve and immediately irradiated as single-cell DMEM suspension at a dose rate of 240 cGy/min for the time required to generate dose curves of 2, 4, 6, 8, and 10Gy with a linear accelerator Clinac 2100C, (Varian Medical Systems, CA, USA), operating at 6 MV. Corresponding controls were sham-irradiated. Colony-forming assays were performed immediately after irradiation by plating cells into triplicate six-well culture dishes. After 21 days, colonies were fixed with 6.0% glutaraldehyde, stained with 0.5% crystal violet, and counted. To generate a radiation survival curve, the surviving fraction (SF) at each radiation dose was normalized to that of the sham-irradiated control, and curves were fitted using a linear-quadratic model, SF. = exp(-αd - βd^2^) in which α is the number of logs of cells killed per gray from the linear portion of the survival curve, and β is the number of logs of cells killed per gray^2 ^from the quadratic component. Three independent experiments were performed.

### Statistical analysis

The approximation of data distribution to normality was preliminarily tested. Results are presented as mean and differences of the means with standard deviations (SDs) or 95% confidence intervals (CI). Statistical analysis was performed using factorial design analysis of variance (ANOVA) and Student two-tailed and unpaired *t *tests as appropriate utilizing Minitab 15™ software. Statistical significance when p < 0.05.

## Results

### A subpopulation of cells with self-renewing and sphere-formation capacity is found among different cell lines established from tumors of the uterine cervix

**Figure 1:**
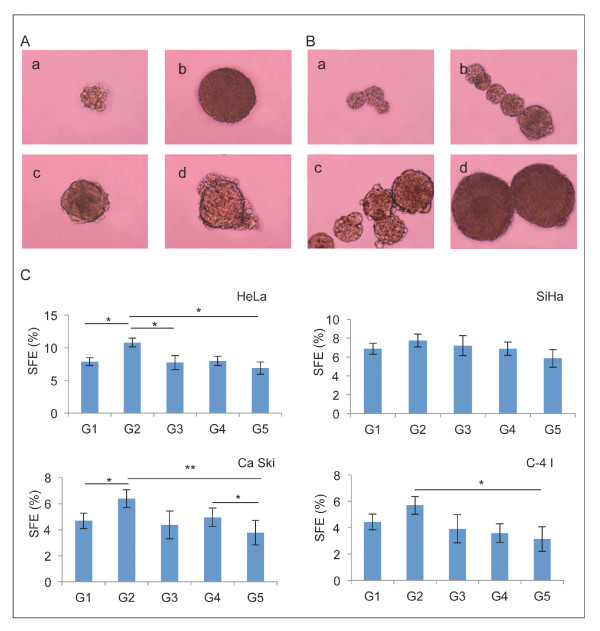
**Spheroids of self-renewing cells from established human cell lines of squamous carcinomas and adenocarcinomas of the cervix**. A, Morphology of cells cultured in anchorage-independent conditions as non-adherent clusters one week after plating HeLa (a), SiHa (b), Ca Ski (c), and C-4 I (d) cells. Magnification is 10X, size bar = 100 μm. B, Serial photographs during culture demonstrated progressive increase diameter at 4 (a), 8 (b), 12 (c) and 16 days (d). Magnification is 10X, size bar = 100 μm. C, Sphere-forming efficiency (SFE) of spheroid cells on serial passage from first to fifth generation (G1-G5). Cells were plated at a density of 100 cells/well in 200-μl of serum-free medium (SFM), spheres were counted weekly. Bar graph represents the SFE mean calculated by counting the number of spheres formed in a given well and dividing by the total number of cells seeded in the well, represented as a percentage. Error bars represent standard deviation (SD) (*n *= 3); * *p *< 0.05 and ** *p *< 0.005

Cells from four well-known cell lines derived from either squamous carcinomas (SiHa, Ca Ski, and C-4 I) or adenocarcinomas (HeLa) of the uterine cervix were cultured at low density (1,000 cells/ml) as suspension in serum-free sphere medium. Six days after plating, nonadherent spherical clusters of cells were observable with different morphologic phenotypes (Figure 1a) similar to spheroids isolated from human primary tumor tissues and cell lines [15,16,19]. All spheroids increased progressively in diameter (Figure 1b). Sphere formation efficiency (SFE) in the first generation was 7.8 ± 0.69% (mean ± SD; *n *= 10 independent experiments) for HeLa, 6.8 ± 0.57% for SiHa, 4.7 ± 0.34% for Ca Ski, and 4.4 ± 0.54% for C-4 I, respectively (Figure 1c). After 7 days in culture primary cervospheres were collected by gentle centrifugation, dissociated, sieved, and re-plated at low density in order to give rise to secondary spheres. SFE was found to be higher in the second generation indicating an increased capacity for sphere formation from passages 1 to 2. Although this enhanced capacity does not persist for several passages spheres could be serially passaged for up to 10 generations and approximately 1-5% of them remained as larger, symmetric, prototypical spheroids providing a definitive evidence for the presence of extended self-renewal. Additionally, when single cells from spheroids were cultured during 7 days under standard differentiating conditions, floating cells could adhere, acquire an epithelial morphology, form symmetric colonies, and survive subsequent passages. As expected, when we examined spheroid-derived cells for CD49f and CD133 CICs markers under differentiating conditions, expression was lost or greatly reduced after 7 days to minimal expression values similar to monolayer cells. Finally, to further support possible differentiation of cell derived from spheres, expression of epithelial differentiation markers was examined demonstrating positivity for cytokeratin AE1/3 in all differentiated spheroids (data not shown). Taken together these data indicate that a subpopulation of cells from spheroids self-renew and differentiate assuming an epithelial tumor phenotype. In addition, to determine whether cells from spheroids possess proliferative potential, we determined their clonogenicity *in vitro*. Cells from spheroids plated at low densities under anchorage-independent conditions showed significantly higher colony-forming abilities compared with their monolayer counterparts. Cells from HeLa, SiHa, Ca Ski, and C-4 I spheroids gave rise to a 1.9-, 1.5-, 2.0- and 2.4-fold greater number of colonies than those from monolayers, respectively. Cells from SiHa spheroids exhibited higher plating efficiency (PE) values (0.95 ± 0.09%) than Hela (0.8 1 ± 0.06%), C-4 I (0.44 ± 0.08%), and Ca Ski (0.42 ± 0.07%) (mean ± SD; *n *= three independent experiments), and these numbers were significantly greater than those obtained from monoloyer cells (*p *< 0.05 and *p *< 0.01), (Additional file 1: Figure S1, available online). These results are consistent with the possibility that cervospheres might be enriched in CCICs.

### Sphere-forming cells express CD49f

**Figure 2:**
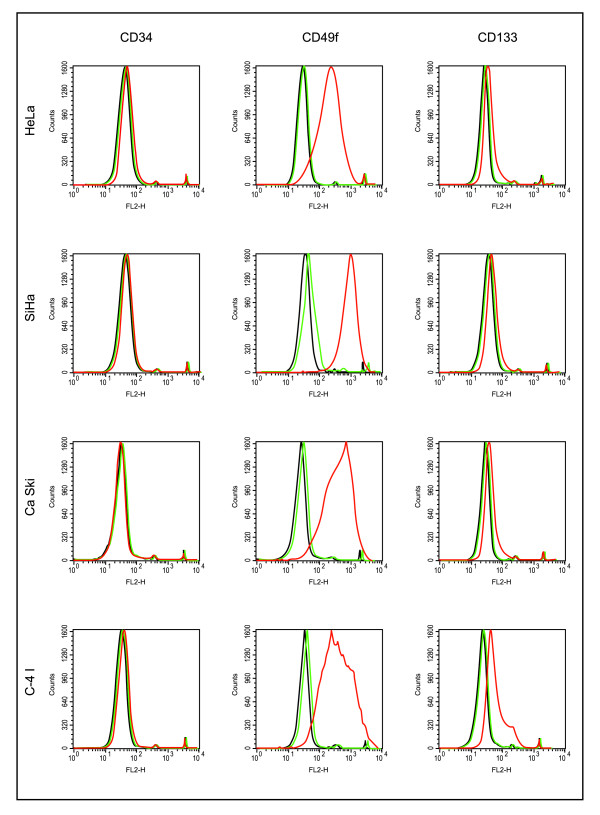
**Immune phenotype analysis of cervical tumor sphere-forming cells**. Sphere-forming cells and monolayer cells were stained with CD34, CD49f, and CD133 conjugated primary antibodies and subjected to flow cytometry. Red line corresponds to sphere-forming cells, green line to monolayer cells, and black line to isotype control

Previous studies showed expression of CD34 as a hematopoietic stem cells marker [6,7], so tested it as the blood and urogenital systems share the same embryonic origin. CD49f has been used as epithelial stem marker in human epidermis [20,21] CD133 is one of the most widely used markers to identify CICs, including epithelial tissues [22]. We performed an initial screen with CD44 and CD71 and found preliminary *in vivo *tumorigenicity assays showed that these populations were not able to generate tumors. We determine whether cultured epithelial cells grown as spheroids are enriched for cells expressing these stem-cell markers. The vast majority of cells stained positive for CD49f, only a minority of them were positive for CD133, while they all showed negative for CD34. Specifically, CD49f-positive cells were 91.67 ± 2.31%, 98.77 ± 3.47%, 92.33 ± 8.39%, and 96.72 ± 4.09% (mean ± SD; *n *= three independent experiments) for HeLa, SiHa, Ca Ski, and C-4 I spheroids cells, respectively. CD133-positive cells were 14.67 ± 4.73%, 9.28 ± 3.15%, 18.36 ± 0.16%, and 61.45 ± 2.26% (mean ± SD; *n *= three independent experiments) for HeLa, SiHa, Ca Ski and C-4 I cells in spheroids. It has been previously shown that sphere-forming cells isolated from primary carcinoma of the cervix uteri [15] and samples from human endometrium [23-25] are negative for CD34. Immunoanalysis of cells growing under adherent or differentiating conditions by flow cytometry demonstrated low frequency levels of these markers, <2% in all cases except for CD49f in SiHa (13.68 ± 2.79%, mean ± SD; *n *= three independent experiments) (Figure 2). These results suggest that sphere-forming CCICs, under stem cell-selective conditions, are enriched for CD49f compared with cervical cell lines and CCICs under differentiating conditions.

### Cervical cancer cell lines harbor Hoechst effluxing cells

**Figure 3:**
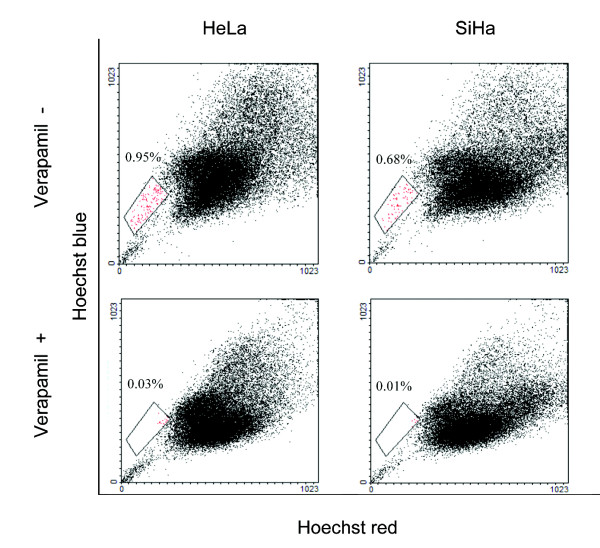
**A side population (SP) is present in HeLa and SiHa cell lines**. Flow cytometry trace from cervical cell preparation incubated for 90 min at 37°C with Hoechst dye as previously described by Goodell et al. (1996). Delimited red region is the (SP) fraction with and without Verapamil

Many stem cells are endowed with the ability to efflux certain lipophilic drugs due to their cell surface expression of ABC family members of membrane transporter proteins and are denominated side population (SP) [18]. When we attempted to explore the existence of a SP within these cancer cell lines, we found that 1.1 ± 0.19% of HeLa (mean ± SD; *n *= 3 independent experiments) and 0.7 ± 0.08% of SiHa cells showed a distinct SP phenotype (Figure 3), confirming that established cell lines from the uterine cervix contain at least two phenotypically distinct sub-populations, one of which bears stem-cell features.

### Sphere-forming cells are more tumorigenic than those grown as monolayer

To investigate the ability of different cell populations to generate tumors, we examined the efficiency of monolayer, sphere-forming, SP, CD34, CD133, and CD49f cells to form tumors in athymic mice, as previously shown for other epithelial CICs [9-12]. SP-positive HeLa cells formed one tumor per two injections with 1 × 10^4 ^cells and CD34 and CD133 positive cells remained nontumorigenic in both cell lines. Injection of 1 × 10^3 ^CD49f^+ ^cells were tumorigenic in 1/6 and 2/6 nude mice, in HeLa and SiHa, respectively. Injections of 1 × 10^3 ^HeLa and SiHa spheroid-derived cells were slightly more tumorigenic in athymic nude mice than CD49f positive cells. 1 × 10^4 ^Ca Ski and C-4 I dissociated spheroid cells allowed formation of tumors, whereas injection of 1 × 10^6 ^cells growth as monolayer remained nontumorigenic (Table 1). HeLa and SiHa cells under adherent conditions were able to produce tumors when at least 5 × 10^5 ^cells were injected, but failed to do so with Ca Ski and C-4 I, albeit with extended latency (10 weeks). Tumors generated with cells from spheroids showed a progressive increase in weight in parallel with the number of injected cells (data not shown). All tumors were readily apparent by visual inspection and palpation, except for a tumor formed with 1 × 10^4 ^SP^+ ^cells, which was detected only upon necropsy. Tumors obtained with cell from spheroids were categorized as poorly differentiated carcinomas (grade 1/grade 2), with histologic features similar to those of the corresponding tumors generated with cell grown as monolayers, including expression of human cytokeratin AE1/3. One essential criterion for CICs is their ability to propagate tumors serially in consecutively engrafted animals. To examine this, serial engraftments of HeLa tumors obtained with cells derived from spheroids were performed by s.c. transplantation of 1-mm tumor pieces into nude mice. Generally, tumors developed 4 weeks after transplantation with a total of four such successful serial transplantations. Secondly, dissociated cells from HeLa tumors obtained with spheroids could reform spheroids under stem cell-selective conditions. Reinjection (s.c.) of 1 × 10^4 ^cells from such secondary sphere-forming cells resulted in tumors with latency that was slightly shorter than that of the parental sphere-forming cells. These results indicate that sphere-forming cells are more malignant than their parental tumor cells, demonstrating that a highly tumorigenic subpopulation of cells resides within cervical cell lines.

**Table 1:** *In vivo *tumorigenicity of cervical cancer-iniciating cells

Subpopulation	Cells	Tumors/injections			
		
		HeLa	SiHa	Ca Ski	C-4 I
	1 × 10^3^	2/6	3/6	0/6	0/6
Spheroid cells	1 × 10^4^	4/6	4/6	3/6	2/6
	1 × 10^5^	5/6	4/6	4/6	4/6
	1 × 10^5^	0/6	0/6	0/6	0/6
	5 × 10^5^	1/6	1/6	0/6	0/6
	1 × 10^6^	5/6	2/6	0/6	0/6

Tumor formation ability of spheroids. Cells were grown in serum-free medium (SFM) as nonadherent cervospheres and assayed for the ability to form tumors after s.c. injection on nu-nu mice at 1 × 10^3^, 1 × 10^4^, and 1 × 10^5 ^cells per injection. At the same time, adherent cells were assayed at 1 × 10^5^, 5 × 10^5^, and 1 × 10^6 ^cells per injection. The number of tumors that formed and the number of injections performed are indicated for each condition

### Spheroid-derived cells exhibit up-regulated expression of CICs genes

**Figure 4:**
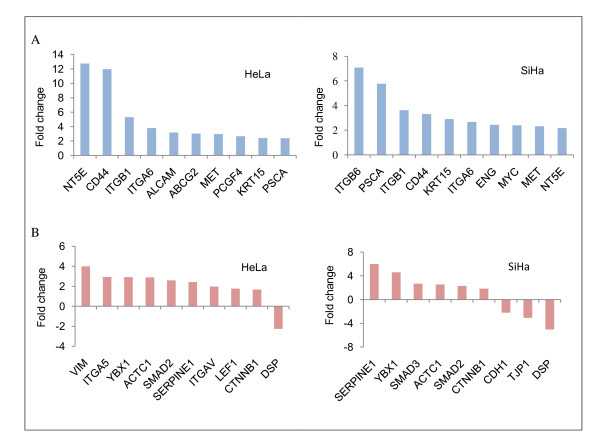
**Cervical cancer-initiating cells (CICs) differentially express characteristic genes of CICs and epithelial to mesenchymal transition (EMT)**. A, Expression of CICs markers in HeLa and SiHa spheroid cells. B, Expression of EMT markers in HeLa and SiHa spheroid cells. Bar graph represents mean fold change (FC) values in spheroid cells compared with those in monolayer, data shown are representative of two independent experiments

**Figure 5:**
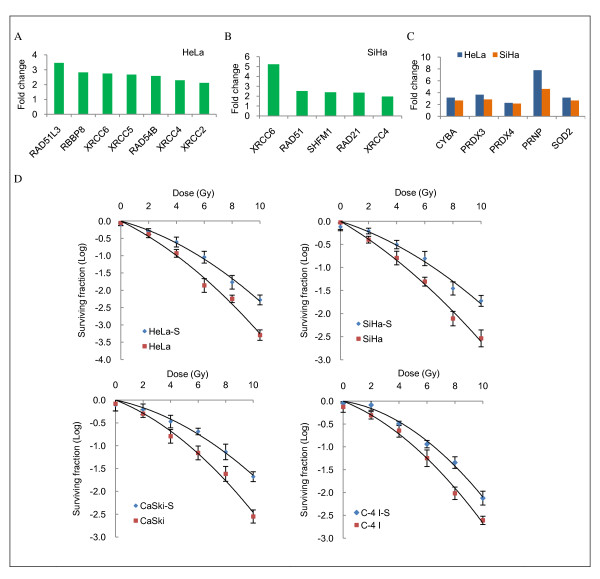
**Cervical cancer-initiating cells (CCICs) are more resistant to X-ray treatment**. A, DNA repair double-strand breaks (DSB) genes differentially expressed in HeLa CCICs. B, as in A but SiHa CICs. C, Reactive oxygen species (ROS) metabolism genes overrepresented in HeLa and SiHa CCICs-enriched spheroids compared with monolayer counterparts. D, Cell survival curves for CCICs-enriched spheroid and monolayer cells. For x-ray treatment, cells were dissociated and irradiated in suspension with 0, 2, 4, 6, 8, and 10 Gy with a linear accelerator Clinac 2100C operating at 200 cGy/min and 6MV. Cells were then seeded in six-well dishes at an appropriate number with DMEM and noble agar (0.3%) for clonogenic survival assay. Twenty one days after irradiation colonies were fixed and stained. Colonies comprising >50 cells were counted for each dose point. To determine surviving fractions (SFs) counts were normalized using the plating efficiency (PE) of the unirradiated corresponding control. Error bars represent standard deviation (SD). Data shown are representative of three independent experiments; S: CCICs-enriched spheroid cells

After demonstration of the ability of tumor-derived cells to organize self-renewing spheroids that possess higher tumorigenic capacity, CICs-specific gene expression was examined. RNA from spheroid or monolayer cells was analyzed by profiling expression with a cDNA microarray. As a result, analysis showed that 767 and 547 genes exhibit a >1.5-fold different expression between HeLa and SiHa cells grown as spheroids versus monolayers, respectively. Stem-cell markers of adult female reproductive system that were up-regulated in cervical spheroids included *CD44 *[15,23,26-28], *ITGB1 *(CD29) [23,24], *PSCA *[29], *NT5E *(CD73) [23-25], *ENG *(CD105) [23,25], *MYC *(c-Myc) [15], *PCGF4 *(BMI-1) [28,30], and *ABCG2 *[28,31-33]. Other epithelial CICs markers found included *ITGB6 *[34], *ALCAM *(CD166) [10,35], *MET *(c-Met) [36], and *KRT15 *[37] (Figure 4a). We were highly interested and indeed found changes in expression of members of other gene groups such as epithelial to mesenchymal transition (EMT)-associated genes (*SERPINE1, YBX1, SMAD3, ACTC1, SMAD2, CTNNB1, CDH1, TJP1, DSP, VIM, ITGA5, ITGAV*, and *LEF1*, Figure 4b); DNA repair double-strand breaks (DSBs) genes (*XRCC6, XRCC5, XRCC4*, and *XRCC2*, non-homologous end-joining genes (NHEJ), and *RAD51, RAD51L3, RBBP8, RAD21, RAD54B*, and *SHFM1*, homologous recombination genes (HR), Figures 5a and 5b) and genes involved in reactive oxygen species (ROS), metabolism (*CYBA, PRDX3, PRDX4, PRNP*, and *SOD2*, Figure 5c). Additional files show description of genes in more detail (Additional files 2,3,4,5,6,&7: Tables S1-S6 and Additional file 8: Figure S2, available online).

### CCICs-enriched spheroids possess a higher degree of radioresistance than monolayer culture-derived cells

To examine whether self-renewing cells from spheroids (exhibiting higher expression of DNA repair and ROS metabolism genes) possess a radioresistant phenotype, we assessed the sensitivity of cervical cell lines to increasing x-ray treatment (up to 10 Gy) under stem cell-selective vs. differentiating conditions. As expected, cervospheres and monolayer cells showed a progressive decrease in survival with an increasing dose of radiation. As shown in Figure 5d, after IR treatment, the SF of the CCICs-enriched population was significantly higher than that of cells grown as monolayer (HeLa monolayer-derived cells, mean SF at 2 Gy (SF_2Gy_) = 0.42 ± 0.06 vs. spheroid cells SF_2Gy _= 0.60 ± 0.11, *p *= 0.05; SiHa monolayer-derived cells, SF_2Gy _= 0.56 ± 0.10 vs. spheroid cells SF_2Gy _= 0.65 ± 0.09, *p *< 0.001; Ca Ski monolayer-derived cells, SF_2Gy _= 0.59 ± 0.04 vs. spheroid cells SF_2Gy _= 0.76 ± 0.06, *p *= 0.02; C-4 I monolayer-derived cells, SF_2Gy _= 0.39 ± 0.05 vs. spheroid cells SF_2Gy _= 0.53 ± 0.02, *p *= 0.02; *n *= 3, two-sided Student *t *test (Table 2). These results support a role for these CICs in cervical cancer radioresistance.

**Table 2:** SF_2_, α and β values in spheres and their monolayer counterparts from established cervical cell lines

	α (Gy^-1^)		β (Gy^-2^)		**SF**_2_	
Cell line	Monolayer	Sphere	Monolayer	Sphere	Monolayer	Sphere
HeLa	0.166 ± 0.044	0.082 ±0.017	0.011 ± 0.006	0.013 ± 0.007	0.42 ± 0.06	0.60 ± 0.11
SiHa	0.099 ± 0.021	0.070 ± 0.011	0.013 ± 0.004	0.016 ± 0.005	0.56 ± 0.10	0.65 ± 0.09
Ca Ski	0.087 ± 0.032	0.032 ± 0.007	0.012 ± 0.000	0.013 ± 0.008	0.59 ± 0.04	0.76 ± 0.06
C-4 I	0.151 ± 0.075	0.115 ± 0.029	0.009 ± 0.003	0.011 ± 0.005	0.39 ± 0.05	0.53 ± 0.02

Parameters of cell survival curves were calculated using a linear-quadratic model for surviving fraction, SF. = exp (-αd - βd^2^). α: number of logs of cells killed per gray from the linear portion of the survival curve; β: number of logs of cells killed per gray^2 ^from the quadratic component. Three independent experiments were performed.

## Discussion

In this report we describe the isolation and characterization of a highly tumorigenic subpopulation of cells from cultures of HeLa, SiHa, Ca Ski, and C-4 I cells that exhibits characteristics of CICs. Previous characterization of CICs from cervical tumor-derived cultures encompassing stem-like cells capable of self-renewal and extensive proliferation was described by Feng et al. [15]. To our knowledge, this is the first characterization of such cells from some of the best-know human cancer cell lines derived from uterine cervix tumors. According to the five separate criteria that have been established to identify candidate CICs [3,13], we found a subpopulation restricted to a small minority of the total cells with self-renewing capacity *in vitro *and *in vivo *(criteria a and b). This subpopulation of sphere-forming cells was more tumorigenic than unselected parental tumor cells and resulted in graft tumors that were histologically identical to the tumors generated from cell lines without selection (criterion c). Differentiation into nontumorigenic cells was evidenced by H&E and cytokeratin staining (criterion d), and cultured spheroid cells exhibited a distinctive cell surface phenotype, CD49f (criterion e). Interestingly, CD49f (*ITGA6*), an alpha-6 integrin, has been postulated as the primary receptor protein in natural human papillomavirus (HPV) infection [38-40]; thus, we postulate that adult reserve cells with expression of CD49f could be preferentially targeted by high-risk HPV types during cervical carcinogenesis.

In addition to CICs markers of the adult female reproductive system, many EMT-associated markers were also found up-regulated in CCICs-enriched spheroids. Recently, it has been reported that induction of EMT in differentiated immortalized human mammary epithelial cells led to the acquisition of the CD44^+^/CD24^- ^stem-cell phenotype [41]. In HeLa, tumorsphere formation and expression of CD44 were significantly elevated when Twist, a key transcriptional factor of EMT, was overexpressed [42]. Our spheroid cells also gained expression of human *ACTC1 *(αSMA) and *VIM *(Vimentin) mesenchymal markers, similar to ovarian cancer cell-formed spheroids [43], and doxorubicin-selected MCF-7/ADR cells [44] indicating that an EMT had occurred during cancer progression of the CICs [45].

Other characteristic of CICs in solid tumors is their radiation resistance, evidenced in breast cancer [46] and glioma tumors [47]. Our functional assay showed that sphere-forming CCICs possess a higher degree of resistance to IR than monolayer cells. We observed increased clonogenic survival for human CCICs-enriched population in which the HR and NHEJ repair pathways and some growth arrest and DNA damage-inducible genes, such as *GADD45A *and *PPP1R15A*, were hyperstimulated, similar to the reported expression of DNA repair-DSBs genes in human embryonic stem cells [48]. Furthermore, Diehn et al. [49], found that expression of genes involved in ROS metabolism, such as *CYBA, SOD2*, and *PRNP*, was highly overrepresented in breast CICs-enriched population. These important antioxidant genes were also enriched in our spheroid cells. Taken together, these findings are consistent with the hypothesis that enhanced expression of ROS defenses and up-regulation of EMT-associated genes [50] in CICs contributes to enhanced cell survival and acquisition of radioresistance.

## Conclusions

We have enriched and characterized a self-renewing subpopulation of CICs among four well known human cancer-derived cell lines (HeLa, SiHa, Ca Ski, and C-4 I), established from squamous carcinomas and adenocarcinomas of the uterine cervix. CICs were enriched as spheroids in selective medium with defined factors and found capable of generation of reproducible tumor phenotypes in nu-nu mice and serial propagation. Injection of 1 × 10^3 ^dissociated spheroid cells allowed formation of tumors in the majority of animals, whereas injection of 1 × 10^5 ^monolayer cells remained nontumorigenic for all cell lines. Sphere-forming CICs expressed CD49f surface marker. When gene profiling analysis of HeLa and SiHa was performed, up-regulation of CICs markers characteristic of the female reproductive system and EMT transition-associated markers was observed. More importantly, this analysis indicated that radioresistance genes were also up-regulated, including components of the DSB-DNA repair machinery and the metabolism of ROS. CICs-enriched populations indeed exhibited an increased resistance to IR. This could be important for addressing and understanding the possible implication of CICs in cancer treatment.

## Abbreviations

bFGF: Basic fibroblast growth factor; BSA: Bovine serum albumin; CCICs: Cervical cancer-initiating cells; CICs: Cancer-initiating cells; DMEM: Dubelcco's modified Eagle's medium; DSBs: Double-strand breaks; EDTA: Ethylene diamine tetraacetic acid; EGF: Epidermal growth factor; EMT: Epithelial to mesenchymal transition; FACS: Fluorescence-activated cell sorting; FBS: Fetal bovine serum; H&E: Hematoxylin and eosin; HPV: Human papillomavirus; HR: Homologous recombination mechanism; IR: Ionizing radiation; MACS: Magnetic-activated cell sorting; NHEJ: Non-homologous end joining mechanism; PE: Phicoeritrin; PE: Plating efficiency; PI: Propidium iodide; ROS: Reactive oxygen species; s.c.: Subcutaneous; SDS: Sodium dodecyl sulfate; SF: Surviving fraction; SFE: Sphere forming-efficiency; SFM: Serum-free medium; SP: Side population; SSC: Saline-sodium citrate.

## Competing interests

The authors declare that they have no competing interests.

## Authors' contributions

JL participated in the acquisition, analysis and interpretation of all data. VMM and CPP conducted cDNA microarray assays and preliminary analysis. AP conducted the radioresistance assay. JL and AGC designed the study and prepared the manuscript. AGC conceived the study and coordinated it. All authors read and approved the final manuscript.

## Pre-publication history

The pre-publication history for this paper can be accessed here:

http://www.biomedcentral.com/1471-2407/12/48/prepub

## Supplementary Material

Additional file 1 **Figure S1**. Plating efficiency for monolayer- and spheroids-derived cells. Click here for file

Additional file 2 **Table S1- Genes**. Selected group of genes whose expression was found up-or down-regulated by a factor of at least 1.5-fold in HeLa spheroid cells compared with HeLa monolayer cells. Click here for file

Additional file 3 **Table S2- Genes**. Selected group of genes whose expression was found up-or down-regulated by a factor of at least 1.5-fold in SiHa spheroid cells compared with SiHa monolayer cells. Click here for file

Additional file 4 **Table S3- Genes**. Biological functions of the genes with altered up-regulated expression by a factor of at least 1.5-fold in HeLa spheroid cells compared with HeLa monolayer cells, as determined by WebGestalt (Gene Set Analysis Toolkit). Click here for file

Additional file 5 **Table S4- Genes**. Biological functions of the genes with altered down-regulated expression by a factor of at least 1.5-fold in HeLa spheroid cells compared with HeLa monolayer cells, as determined by WebGestalt (Gene Set Analysis Toolkit). Click here for file

Additional file 6 **Table S5- Genes**. Biological functions of the genes with altered up-regulated expression by a factor of at least 1.5-fold in SiHa spheroid cells compared with SiHa monolayer cells, as determined by WebGestalt (Gene Set Analysis Toolkit). Click here for file

Additional file 7 **Table S6- Genes**. Biological functions of the genes with altered down-regulated expression by a factor of at least 1.5-fold in SiHa spheroid cells compared with SiHa monolayer cells, as determined by WebGestalt (Gene Set Analysis Toolkit). Click here for file

Additional file 8 **Figure S2- Genes**. Vein diagram for common genes between HeLa and SiHa spheroid cells compared with monolayer cells, whose expression was found up- and down-regulated by a factor of at least 1.5-fold. Click here for file
