# Supplementary material for: Cancer-initiating cells derived from established cervical cell lines exhibit stem-cell markers and increased radioresistance
Source: BMC Cancer. 2012 Jan 28;12:48. doi: 10.1186/1471-2407-12-48 (PMC3299592; doi:10.1186/1471-2407-12-48)

Supplementary Figure 1. Plating efficiency for monolayer- and spheroids-derived cells. Bar graph represents the platting efficiency mean calculated by counting the number of colonies formed in a given well and dividing by the total number of cells seeded in the well, represented as a percentage. Error bars represent standard deviation (SD) ($n = 3$); * $p < 0.05$ and ** $p < 0.005$.

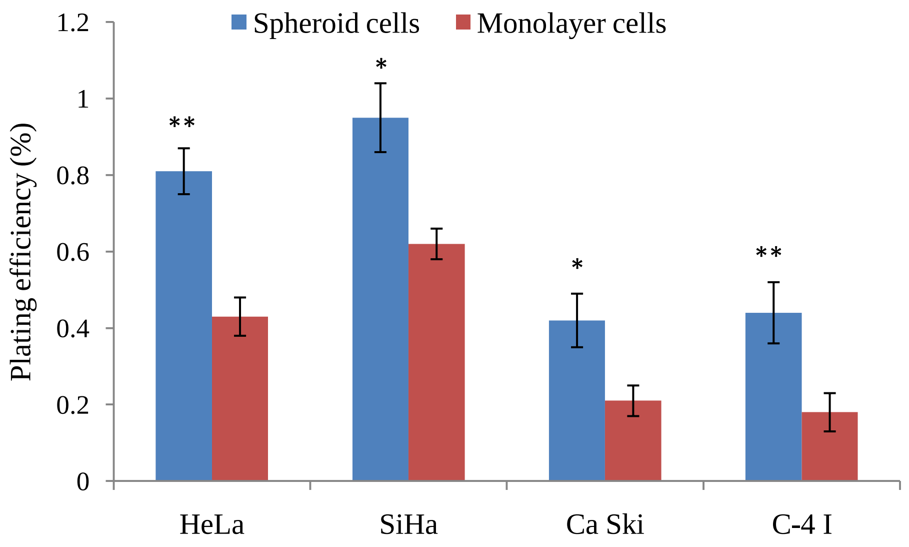

Supplement: Additional file 1 — Figure S1. Plating efficiency for monolayer- and spheroids-derived cells. [file 1471-2407-12-48-S1.PDF]
