# Supplementary material for: Cancer-initiating cells derived from established cervical cell lines exhibit stem-cell markers and increased radioresistance
Source: BMC Cancer. 2012 Jan 28;12:48. doi: 10.1186/1471-2407-12-48 (PMC3299592; doi:10.1186/1471-2407-12-48)
Supplement: Additional file 2 — Table S1- Genes. Selected group of genes whose expression was found up-or down-regulated by a factor of at least 1.5-fold in HeLa spheroid cells compared with HeLa monolayer cells. [file 1471-2407-12-48-S2.PDF]

Supplementary Table 1. Selected group of genes whose expression was found up- or down-regulated by a factor of at least 1.5-fold in HeLa spheroid cells compared with HeLa monolayer cells.

Cancer-initiating cells (CICs)-associated genes

**Official symbol**	**Official full name**	**Genbank account no.**	**Fold change**
CD44	CD44 molecule (Indian blood group)	NM_000610.3	11.95
ITGB1	Integrin, beta 1 (fibronectin receptor, beta polypeptide, antigen CD29 includes MDF2, MSK12)	NM_002211.3	5.29
PSCA	Prostate stem cell antigen	NM_005672.4	2.37
NT5E	5'-nucleotidase, ecto (CD73)	NM_002526.3	12.73
PCGF4	BMI1 polycomb ring finger oncogene	NM_005180.8	2.66
ABCG2	ATP-binding cassette, sub-family G (WHITE), member 2	NM_004827.2	3.02
ALCAM	Activated leukocyte cell adhesion molecule	NM_001627.2	3.17
MET	Met proto-oncogene (hepatocyte growth factor receptor)	NM_001127500.1	2.94
ITGA6	Integrin, alpha 6	NM_001079818.1	3.79
KRT15	Keratin 15	NM_002275.3	2.40

Epithelial to mesenchymal transition (EMT)-associated genes

Official symbol	Official full name	Genbank account no.	Fold change
SERPINE1	Serpin peptidase inhibitor, clade E (nexin, plasminogen activator inhibitor type 1), member 1	NM_000602.3	2.43
YBX1	Y box binding protein 1	NM_004559.3	2.91
ACTC1	Actin, alpha, cardiac muscle 1	NM_005159.4	2.88
SMAD2	SMAD family member 2	NM_005901.4	2.59
CTNNB1	Catenin (cadherin-associated protein), beta 1, 88kDa	NM_001904.3	1.67
DSP	Desmoplakin	NM_004415.2	-2.25
VIM	Vimentin	NM_003380.3	3.99
ITGA5	Integrin, alpha 5 (fibronectin receptor, alpha polypeptide)	NM_002205.2	2.92
ITGAV	Integrin, alpha V (vitronectin receptor, alpha polypeptide, antigen CD51)	NM_002210.3	1.96
LEF1	Lymphoid enhancer-binding factor 1	NM_016269.4	1.76

Non-homologous end-joining (NHEJ)-associated genes

**Official symbol**	**Official full name**	**Genbank account no.**	**Fold change**
XRCC6	X-ray repair complementing defective repair in Chinese hamster cells 6	NM_001469.3	2.75
XRCC5	X-ray repair complementing defective repair in Chinese hamster cells 5	NM_021141.3	2.67
XRCC4	X-ray repair complementing defective repair in Chinese hamster cells 4	NM_003401.3	2.29
XRCC2	X-ray repair complementing defective repair in Chinese hamster cells 2	NM_005431.1	2.11

Homologous recombination (HR)-associated genes

**Official symbol**	**Official full name**	**Genbank account no.**	**Fold change**
RAD51L3	RAD51-like 3	NM_002878.3	3.46
RBBP8	Retinoblastoma binding protein 8	NM_002894.2	2.82
RAD54B	RAD54 homolog B	NM_012415.3	2.57

Metabolism of reactive oxygen species (ROS)-associated genes

**Official symbol**	**Official full name**	**Genbank account no.**	**Fold change**
CYBA	Cytochrome b-245, alpha polypeptide	NM_000101.2	3.16
PRDX3	Peroxiredoxin 3	NM_006793.2	3.66
PRDX4	Peroxiredoxin 4	NM_006406.1	2.27
PRNP	Prion protein	NM_000311.3	7.80
SOD2	Superoxide dismutase 2	NM_000636.2	3.16
