# Supplementary material for: Cancer-initiating cells derived from established cervical cell lines exhibit stem-cell markers and increased radioresistance
Source: BMC Cancer. 2012 Jan 28;12:48. doi: 10.1186/1471-2407-12-48 (PMC3299592; doi:10.1186/1471-2407-12-48)
Supplement: Additional file 3 — Table S2- Genes. Selected group of genes whose expression was found up-or down-regulated by a factor of at least 1.5-fold in SiHa spheroid cells compared with SiHa monolayer cells. [file 1471-2407-12-48-S3.PDF]

Supplementary Table 2. Selected group of genes whose expression was found up- or down-regulated by a factor of at least 1.5-fold in SiHa spheroid cells compared with SiHa monolayer cells.

Cancer-initiating cells (CICs)-associated genes

**Official symbol**	**Official full name**	**Genbank account no.**	**Fold change**
CD44	CD44 molecule (Indian blood group)	NM_000610.3	3.31
ITGB1	Integrin, beta 1 (fibronectin receptor, beta polypeptide, antigen CD29 includes MDF2, MSK12)	NM_002211.3	3.61
PSCA	Prostate stem cell antigen	NM_005672.4	5.77
NT5E	5'-nucleotidase, ecto (CD73)	NM_002526.3	2.17
ENG	Endoglin	NM_001114753.1	2.44
MYC	v-myc myelocytomatosis viral oncogene homolog	NM_002467.4	2.39
ITGB6	Integrin, beta 6	NM_000888.3	7.08
MET	Met proto-oncogene (hepatocyte growth factor receptor)	NM_001127500.1	2.32
ITGA6	Integrin, alpha 6	NM_001079818.1	2.66
KRT15	Keratin 15	NM_002275.3	2.90

Epithelial to mesenchymal transition (EMT)-associated genes

Official symbol	Official full name	Genbank account no.	Fold change
SERPINE1	Serpin peptidase inhibitor, clade E (nexin, plasminogen activator inhibitor type 1), member 1	NM_000602.3	5.99
YBX1	Y box binding protein 1	NM_004559.3	4.58
SMAD3	SMAD family member 3	NM_005902.3	2.67
ACTC1	Actin, alpha, cardiac muscle 1	NM_005159.4	2.53
SMAD2	SMAD family member 2	NM_005901.4	2.28
CTNNB1	Catenin (cadherin-associated protein), beta 1, 88kDa	NM_001904.3	1.82
CDH1	Cadherin 1, type 1, E-cadherin (epithelial)	NM_004360.3	-2.21
TJP1	Tight junction protein 1 (zona occludens 1)	NM_003257.3	-3.08
DSP	Desmoplakin	NM_004415.2	-5.04
VIM	Vimentin	NM_003380.3	

Non-homologous end-joining (NHEJ)-associated genes

Official symbol	Official full name	Genbank account no.	Fold change
XRCC6	X-ray repair complementing defective repair in Chinese hamster cells 6	NM_001469.3	5.24
XRCC4	X-ray repair complementing defective repair in Chinese hamster cells 4	NM_003401.3	1.97

Homologous recombination (HR)-associated genes

Official symbol	Official full name	Genbank account no.	Fold change
RAD51	RAD51 homolog	NM_002875.4	2.52
RAD21	RAD21 homolog	NM_006265.2	2.35
SHFM1	Split hand/foot malformation (ectrodactyly) type 1	NM_006304.1	2.39

Metabolism of reactive oxygen species (ROS)-associated genes

Official symbol	Official full name	Genbank account no.	Fold change
CYBA	Cytochrome b-245, alpha polypeptide	NM_000101.2	2.67
PRDX3	Peroxiredoxin 3	NM_006793.2	2.86
PRDX4	Peroxiredoxin 4	NM_006406.1	2.15
PRNP	Prion protein	NM_000311.3	4.63
SOD2	Superoxide dismutase 2	NM_000636.2	2.67
