# Supplementary material for: Cancer-initiating cells derived from established cervical cell lines exhibit stem-cell markers and increased radioresistance
Source: BMC Cancer. 2012 Jan 28;12:48. doi: 10.1186/1471-2407-12-48 (PMC3299592; doi:10.1186/1471-2407-12-48)
Supplement: Additional file 4 — Table S3- Genes. Biological functions of the genes with altered up-regulated expression by a factor of at least 1.5-fold in HeLa spheroid cells compared with HeLa monolayer cells, as determined by WebGestalt (Gene Set Analysis Toolkit). [file 1471-2407-12-48-S4.PDF]

Supplementary Table 3. Biological functions of the genes with altered up-regulated expression by a factor of at least 1.5-fold in HeLa spheroid cells compared with HeLa monolayer cells, as determined by WebGestalt (Gene Set Analysis Toolkit).

Category	Function	Gene Symbol	No. of molecules
Biological Process	Translation	KARS, RPL26, RPL9, MRPL42, MTIF2, EIF1AX, RPL6, EIF4G2, RPL5, EEF1A1, PAIP1, RPS3A, DENR, EEF1B2, IGF2BP3, SRP9, EIF2S2, IARS, RPS7, EIF2S3, ITGA2, DARS2, SELT, MRPL1, RPL26L1, EEF1E1, MRPL3	27
Biological Process	Metabolic Process	PTP4A1, ST13, EIF1AX, NPM1, PPIL1, GLUD1, EXT1, NT5C3, SLC20A1, MOCS2, PRNP, MTRR, F3, SNX6, CYP51A1, AASDHPPT, SNRPD1, EDEM1, LMAN2L, MAN2A1, ARNT2, TRAPPC2, SMARCE1, PTPRK, UBE2F, TRNT1, CD55, RPL9, ZNF195, OSBP2, SKIV2L2, PBK, GMFB, ZNF673, CAV1, SERPINB4, MCFD2, ODC1, LIPA, TTRAP, GNAI3, CBFB, PLCXD2, HSPE1, PDSS1, ATP5F1, TAF9B, SMN2, TRMT5, OGT, MRPL1, ZC3H8, FBXL3, HDAC2, NDUFV2, HMGB1, SERPINE2, PPP1R2, POP4, TGFBR2, RPL5, IGF2BP3, RNF141, SUB1, EIF2S2, LSM5, MSH2, RFX5, UBE3C, SC5DL, CASP1, PPA1, SERBP1, ASF1A, NT5E, TRAK2, SMS, IMPA1, CYP26B1, SELI, RPL26, GTF2H3, CREG1, ELL2, SF3B14, RDH10, ERO1L, RPL6, PSMA1, MAP4K3, UFM1, FRG1, EIF4G2, CYB5B, HMGN2, TFF1, SRP9, PTPRF, ADSS, NT5C2, NAP1L1, SYNCRIP, HIF1A, RBMX, PRPS2, GNPAT, C2orf3, SLC16A1, UMPS, SMARCA5, PNPT1, MRPL3, CCNE2, PLA2G4A, NDUFC2, SPRED1, KARS, SQRDL, DUSP6, PSMA2, BZW1, FTH1, PUS7, PHLDA1, PAIP1, DENR, EEF1B2, CD46, PSMA6, TDG, AHCTF1, ASNS, CAP1, DUS4L, IARS, CBR4, UCHL5, SOCS6, ACSL4, LMAN1, EGLN3, RPL26L1, F2R, PRMT3, IGFBP3, SOAT1, C1D, MTIF2, MTMR6, YWHAQ, MRPS35, GLO1, ZMPSTE24, SDHD, EDN2, UBE2V2, RPS7, RP6-213H19.1, ITGA2, C1orf27, ADAT1, DARS2, PTPLAD1, PPP1CB, TAF9, PPIL3, ECHDC1, EEF1E1, UBE2N, CDC40, INSIG2, MRPL42, DMD, UTP14A, SLC25A12, SPCS2, RPE,	223

		RBL2, SCYL2, EEF1A1, ADH5, TTC1, PHTF2, CTSC, PGM3, LYPLA1, SELT, XRN2, WWP1, CSDA, ITGA1, PRPF40A, STAMBPL1, COPS2, PSAT1, POLK, SMARCAD1, GCLC, GATAD1, GGH, MTMR12, IFNAR1, TGDS, RPS3A, POLR3G, TIMP3, PRDX3, PGK1, EIF2S3, NEIL3, TPRKB, ORC5L, ZNF277, RYK, RNF138, GLRX, SFRS3, CACYBP, PSME3, NDFIP1, PTGES, GCSH, USP16	
Biological Process	Cellular Metabolic Process	PTP4A1, ST13, EIF1AX, NPM1, PPIL1, GLUD1, EXT1, NT5C3, SLC20A1, MOCS2, MTRR, SNX6, CYP51A1, SNRPD1, EDEM1, LMAN2L, MAN2A1, ARNT2, TRAPPC2, SMARCE1, PTPRK, UBE2F, TRNT1, RPL9, ZNF195, OSBP2, SKIV2L2, PBK, GMFB, ZNF673, CAV1, MCFD2, ODC1, LIPA, TTRAP, GNAI3, CBFB, HSPE1, PDSS1, ATP5F1, TAF9B, SMN2, TRMT5, OGT, MRPL1, ZC3H8, FBXL3, HDAC2, NDUFV2, HMGB1, PPP1R2, POP4, TGFBR2, RPL5, IGF2BP3, RNF141, SUB1, EIF2S2, LSM5, MSH2, RFX5, UBE3C, SC5DL, PPA1, SERBP1, ASF1A, NT5E, TRAK2, SMS, IMPA1, CYP26B1, SELI, RPL26, GTF2H3, CREG1, ELL2, SF3B14, ERO1L, RPL6, PSMA1, MAP4K3, UFM1, FRG1, EIF4G2, CYB5B, HMGN2, SRP9, PTPRF, ADSS, NT5C2, NAP1L1, SYNCRIP, HIF1A, RBMX, PRPS2, GNPAT, C2orf3, SLC16A1, UMPS, SMARCA5, PNPT1, MRPL3, CCNE2, PLA2G4A, NDUFC2, SPRED1, KARS, DUSP6, PSMA2, BZW1, PUS7, PHLDA1, PAIP1, DENR, EEF1B2, PSMA6, TDG, AHCTF1, ASNS, CAP1, DUS4L, IARS, UCHL5, SOCS6, ACSL4, LMAN1, RPL26L1, F2R, IGFBP3, SOAT1, C1D, MTIF2, TMR6, YWHAQ, MRPS35, GLO1, ZMPSTE24, SDHD, EDN2, UBE2V2, RPS7, RP6-213H19.1, ITGA2, ADAT1, DARS2, PTPLAD1, PPP1CB, TAF9, PPIL3, EEF1E1, UBE2N, CDC40, INSIG2, MRPL42, DMD, UTP14A, SLC25A12, SPCS2, RBL2, SCYL2, EEF1A1, ADH5, TTC1, PHTF2, PGM3, LYPLA1, SELT, XRN2, WWP1, CSDA, ITGA1, PRPF40A, STAMBPL1, COPS2, PSAT1, POLK, SMARCAD1, GCLC, GATAD1, GGH, MTMR12, IFNAR1, TGDS, RPS3A, POLR3G, TIMP3, PRDX3, PGK1, EIF2S3, NEIL3, ORC5L, ZNF277, RYK, RNF138, GLRX, SFRS3, CACYBP, PSME3, NDFIP1, PTGES, GCSH, USP16	202
Biological Process	Translational	SRP9, RPL26, RPL9, RPS7, RPL6, SELT, RPL26L1, EEF1A1, RPL5,	11

	elongation	RPS3A, EEF1B2	
Biological Process	Cellular protein metabolic process	PTP4A1, KARS, ST13, DUSP6, PSMA2, EIF1AX, PPIL1, MOCS2, PAIP1, DENR, EEF1B2, PSMA6, IARS, EDEM1, LMAN2L, UCHL5, SOCS6, MAN2A1, LMAN1, RPL26L1, F2R, IGFBP3, PTPRK, UBE2F, RPL9, PBK, MTIF2, GMFB, MTMR6, CAV1, LIPA, HSPE1, ZMPSTE24, UBE2V2, RPS7, RP6-213H19.1, ITGA2, DARS2, OGT, PTPLAD1, PPP1CB, MRPL1, TAF9, FBXL3, PPIL3, EEF1E1, UBE2N, HDAC2, MRPL42, SPCS2, TGFBR2, SCYL2, EEF1A1, RPL5, IGF2BP3, ADH5, TTC1, EIF2S2, SELT, WWP1, ITGA1, UBE3C, TRAK2, RPL26, STAMBPL1, GCLC, ERO1L, RPL6, PSMA1, MAP4K3, UFM1, EIF4G2, RPS3A, PTPRF, SRP9, TIMP3, EIF2S3, RYK, RNF138, CACYBP, PSME3, NDFIP1, MRPL3, USP16	84
Biological process	RNA processing	RPL26, TRNT1, CDC40, C1D, SKIV2L2, UTP14A, SF3B14, NPM1, PPIL1, POP4, PUS7, FRG1, RPL5, SNRPD1, DUS4L, SYNCRIP, LSM5, SMN2, TRMT5, RPS7, RBMX, ADAT1, XRN2, SFRS3, MRPL1, PPIL3, PNPT1, PRPF40A	28
Biological process	Healing during inflammatory response	CD44, F2R, HIF1A	3
Biological process	Translational initiation	EIF2S2, EIF2S3, MTIF2, EIF1AX, EIF4G2, PAIP1, DENR, RPS3A	8
Biological process	Regulation of anatomical structure size	GCLC, DSTN, ACTR3, NPM1, TMEM123, CAV1, CAPZA2, NTS, TAF9B, DCBLD2, EDN2, TMSL3, TMSL2, CAPZA1, XRN2, CD44, ITGA1, TAF9, NRP1, F2R, IGFBP3	21
Biological process	Response to stress	PLA2G4A, HMGB1, INSIG2, NPM1, TLR10, PRNP, F3, ADH5, CD46, ASNS, TDG, HIGD1A, EDEM1, KCNN4, PRKRIR, SOCS6, MSH2, ARNT2, CD44, LMAN1, EGLN3, ERRFI1, CSDA, F2R, CASP1, ASF1A, NT5E, PTPRK, FAIM3, GTF2H3, CD55, CYSLTR1, POLK,GCLC, ERO1L, MRPS35, MAP4K3, CAV1, LIPA, TTRAP, ANGPTL4, HSPE1, POLR3G, DCBLD2, TFF1, TIMP3, UBE2V2, HIF1A, PRDX3, NEIL3, ITGA2, IL8, PTPLAD1, ANXA2, NRP1, TAF9, NDFIP1, EEF1E1, UBE2N, HDAC2	
Molecular function	RNA binding	EIF1AX, NPM1, POP4, PUS7, RPL5, PAIP1, IGF2BP3, PSMA6,	36

		DAZAP1, SNRPD1, EIF2S2, LSM5, SERBP1. SRP72, TRNT1, RPL26, RPL9, C1D, STAU2, SF3B14, RPL6, PSMA1, XPOT, RPS3A, THUMPD3, SRP9, SMN2, SYNCRIP, RPS7, RBMX, ADAT1, SFRS3, MRPL1, DDX10, MRPL3, PNPT1	
Molecular function	Protein binding	ST13, ACTG1, LGR4, BTBD7, NPM1, GLUD1, EXT1, MOCS2, PRNP, F3, SNX6, AASDHPPT, SNRPD1, SNX4, EDEM1, TMSL2, ARNT2, TRAPPC2, SMARCE1, STC1, PTPRK, UBE2F, ACTR6, SKIV2L2, PBK, DSTN, AP1S2, GMFB, CAV1, SERPINB4, ODC1, NTS, TTRAP, GNAI3, CBFB, HSPE1, PDSS1, ATP5F1, TAF9B, SASH1, SMN2, TMED10, LRRC40, IL8, OGT, ACTB, NRP1, FBXL3, HDAC2, NXT2, HMGB1, PPP1R2, TXNDC9, POP4, TGFBR2, RPL5, MYO5C, IGF2BP3, RNF141, SUB1, EIF2S2, LSM5, KCNN4, COPS3, COL6A1, CAPZA1, MSH2, RFX5, UBE3C, CASP1, CAV2, TMED2, SERBP1, ASF1A, TRAK2, IMPA1, RPL26, GTF2H3, SH2D2A, CREG1, SF3B14, ERO1L, PSMA1, MAP4K3, SNAP25, CAPZA2, UFM1, LIN7C, EIF4G2, ANGPTL4, THUMPD3, HMGN2, BCAR3, TFF1, SRP9, PTPRF, NT5C2, NAP1L1, SYNCRIP, HIF1A, RBMX, CD47, GNPAT, C2orf3, SLC16A1, MAL2, BTBD10, SMARCA5, CCNE2, SPRED1, TOMM20, SCAMP1, DUSP6, PSMA2, BZW1, ACTR3, FTH1, ACTG2, TLR10, PHLDA1, PAIP1, EEF1B2, PHC2, PSMA6, TDG, ASNS, D164, CAP1, KCTD16, HIGD1A, ABCD3, UCHL5, PRKRIR, SOCS6, APIP, LMAN1, EGLN3, F2R, PERP, IGFBP3, C1D, OSTM1, YWHAQ, C14orf166, SMYD2, LXN, MAGI3, DCBLD2, EDN2, UBE2V2, RPS7, RP6-213H19.1, ITGA2, LAMB1, PTPLAD1, PPP1CB, GRB10, ANXA2, TAF9, PPIL3, EEF1E1, UBE2N, HIATL1, INSIG2, DMD, UTP14A, SLC25A12, RPE, RBL2, EEF1A1, CDR2, VPS26A, CD58, ADH5, TTC1, CLIC4, ADD3, TMSL3, CBWD2, CTSC, XRN2, WWP1, CD44, ITGA1, CSDA, ERRFI1, C15orf41, MPHOSPH6, PRPF40A, RIN2, COPS2, CTNNAL1, GCLC, SMARCAD1, PDE2A, IFNAR1, TGDS, RNF13, RPS3A, TIMP3, TWSG1, PRDX3, EIF2S3, TPRKB, ORC5L, ZNF277, RYK, RNF138, GLRX, SFRS3, CACYBP, PSME3, NDFIP1, USP16	214
Molecular function	Translation factor activity, nucleic	GTF2H3, EIF2S2, EIF2S3, MTIF2, EIF1AX, EIF4G2, EEF1A1, EEF1B2, DENR	9

	acid binding		
Molecular function	Nitric-oxide synthase binding	ACTB, CAV1, DMD	3
Molecular function	5'-nucleotidase activity	NT5C2, NT5C3, NT5E	3
Molecular function	Binding	ACTG1, LGR4, EIF1AX, NPM1, GLUD1, NT5C3, MOCS2, SNX6, SNRPD1, SNX4, EDEM1, MAN2A1, TRAPPC2, SMARCE1, STC1, UBE2F, SRP72, TRNT1, RPL9, PBK, DSTN, AP1S2, ZNF673, SERPINB4, MCFD2, TTRAP, GNAI3, CBFB, HSPE1, SASH1, TMED10, LRRC40, OGT, MRPL1, FBXL3, NRP1, HDAC2, NXT2, NDUFV2, HMGB1, ATAD1, TXNDC9, POP4, TGFBR2, IGF2BP3, ANXA10, EIF2S2, COPS3, AIM1, MSH2, CASP1, PPA1, CAV2, TMED2, SERBP1, ASF1A, TRAK2, RPL26, GTF2H3, TEX10, SF3B14, RDH10, ERO1L, SNAP25, CAPZA2, ANGPTL4, HMGN2, ADSS, PTPRF, SRP9, NT5C2, HIF1A, RBMX, GNPAT, C2orf3, MAL2, SMARCA5, MRPL3, PLA2G4A, SPRED1, KARS, SCAMP1, DUSP6, BZW1, ACTR3, FTH1, PHC2, TDG, AHCTF1, ASNS, CD164, ABCD3, UCHL5, SOCS6, ACSL4, PERP, C1D, MTIF2, SMYD2, XPOT, MAGI3, ZMPSTE24, SDHD, DCBLD2, AMZ2, RPS7, RP6-213H19.1, LAMB1, C1orf27, PTPLAD1, PPP1CB, GRB10, ANXA2, TAF9, UBE2N, INSIG2, DMD, UTP14A, SLC25A12, RPE, RBL2, EEF1A1, CD58, PHTF2, CLIC4, ADD3, CBWD2, CTSC, XRN2, WWP1, ERRFI1, CSDA, ITGA1, C15orf41, MPHOSPH6, PRPF40A, STAMBPL1, PSAT1, CTNNAL1, POLK, TIMP3, EIF2S3, PGK1, PRDX3, TPRKB, ORC5L, RNF138, RYK, GLRX, PSME3, USP16, ST13, BTBD7, SMOC1, EXT1, F3, MTRR, PRNP, CYP51A1, AASDHPPT, NT5DC1, LMAN2L, TMSL2, ARNT2, SLC39A10, PTPRK, ACTR6, ZNF195, SKIV2L2, STAU2, GMFB, ARMC1, CAV1, NTS, ODC1, TAF9B, ATP5F1, PDSS1, PI4K2B, SMN2, IL8, ACTB, GNL3L, ZC3H8, PPP1R2, SERPINE2, RPL5, MYO5C, RNF141, SUB1, C1GALT1, TSNAX, DAZAP1, LSM5, KCNN4, COL6A1, CAPZA1, RFX5, UBE3C, SC5DL, NT5E, CYP26B1, FOLR3, IMPA1, SELI, CREG1, SH2D2A, RPL6, PSMA1, MAP4K3, UFM1, CYB5B, EIF4G2, LIN7C, THUMPD3, TFF1, BCAR3, NAP1L1,	295

		SYNCRIP, CD47, PRPS2, RAB31, SLC16A1, BTBD10, DDX10, PNPT1, ZFAND1, CCNE2, TOMM20, PSMA2, TLR10, ACTG2, PUS7, PHLDA1, PAIP1, EEF1B2, DENR, PSMA6, IARS, DUS4L, CAP1, HIGD1A, KCTD16, CBR4, PRKRIR, APIP, EGLN3, LMAN1, F2R, PRMT3, IGFBP3, SOAT1, OSTM1, YWHAQ, GLO1, C14orf166, LXN, EDN2, UBE2V2, ITGA2, ADAT1, DARS2, PPIL3, EEF1E1, ANXA3, HIATL1, SPOCK1, SCYL2, CDR2, VPS26A, ADH5, TTC1, TMSL3, PGM3, FAM82B, SELT, CD44, RIN2, COPS2, GCLC, SMARCAD1, GATAD1, PDE2A, IFNAR1, TGDS, RNF13, RPS3A, TMEM38B, TWSG1, NEIL3, ZNF277, SFRS3, CACYBP, NDFIP1, GCSH	
Molecular function	Nucleotidase activity	NT5C2, NT5C3, NT5E	3
Molecular function	Magnesium ion binding	IMPA1, SELI, TRNT1, POLK, GCLC, NT5C3, TTRAP, TGFBR2, ADSS, NT5C2, C1GALT1, AASDHPPT, RP6-213H19.1, NT5DC1, ITGA2, PRPS2, PGM3, MSH2, ACSL4, ITGA1, PPA1	21
Molecular function	Translation initiation factor activity	EIF2S2, EIF4G2, EIF2S3, MTIF2, EIF1AX, DENR	6
Molecular function	Coenzyme binding	ADH5, SOAT1, NDUFV2, DUS4L, GCLC, CBR4, ERO1L, GLUD1, TGDS, GCSH, MTRR	11
Cellular component	Cytoplasm	PTP4A1, TMED7, ST13, ACTG1, EIF1AX, NPM1, GLUD1, EXT1, NT5C3, MOCS2, PRNP, MTRR, SNX6,, CYP51A1, AASDHPPT, SNRPD1, YIPF4, SNX4, EDEM1, LMAN2L, TMSL2, MAN2A1, COX4NB, TRAPPC2, SRP72, FAIM3, ACTR6, TRNT1, RPL9, GPRC5A, STAU2, DSTN, AP1S2, CAV1, SERPINB4, MCFD2 ODC1, NTS, LIPA, GNAI3, HSPE1, ATP5F1, PI4K2B, SMN2, TRMT5, TMED10, OGT, ACTB, MRPS28, MRPL1, FBXL3, HDAC2, NXT2, NDUFV2, RPL5, IGF2BP3, ANXA10, DAZAP1, TSNAX, EIF2S2, COPS3, SC5DL, CASP1, PPA1, CAV2, TMED2, SERBP1, NT5E, TRAK2, SMS, IMPA1, CYP26B1, RPL26, MMD, SH2D2A, RDH10, ERO1L, RPL6, PSMA1, VEZT, SNAP25, CAPZA2, UFM1, EIF4G2, CYB5B, HMGN2, SRP9, PTPRF, ADSS, NT5C2, NAP1L1, SYNCRIP, HIF1A, GNPAT, UMPS, MAL2, FAM5C, PNPT1, MRPL3, CCNE2, PLA2G4A, ASAM, NDUFC2,	208

		KARS, TOMM20, SQRDL, SCAMP1, DUSP6, PSMA2, BZW1, ACTR3, FTH1, PLEK2, ACTG2, PHLDA1, PAIP1, EEF1B2, MRPL50, PSMA6, AHCTF1, ASNS, CD164, CAP1, IARS, ABCD3, CBR4, SOCS6, APIP, ACSL4, LMAN1, EGLN3, RPL26L1, F2R, PRMT3, PERP, SOAT1, C1D, OSTM1, MTIF2, MTMR6, YWHAQ, MRPS35, GLO1, C14orf166, LXN, XPOT, ZMPSTE24, SDHD, UBE2V2, RPS7, RP6, 213H19.1, ITGA2, LAMB1, DARS2, PPP1CB, GRB10, ANXA2, EEF1E1, UBE2N, ANXA3, INSIG2, MRPL42, DMD, SLC25A12, SPCS2, CNIH, RPE, SCYL2, EEF1A1, CDR2, VPS26A, ADH5, MED5, PHTF2, CLIC4, ADD3, TMSL3, CTSC, FAM82B, LYPLA1, CD44, ITGA1, CSDA, ERRFI1, MPHOSPH6, RIN2, COPS2, CTNNAL1, GCLC, GGH, MTMR12, TMEM50A, FNDC3A, RNF13, RPS3A, TIMP3, TMEM38B, PRDX3, PGK1, EIF2S3, TPRKB, GLRX, CACYBP, PSME3, NDFIP1, PTGES, GCSH, USP16	
Cellular component	Intracellular part	PTP4A1, TMED7, ACTG1, EIF1AX, NPM1, GLUD1, NT5C3, MOCS2, SNX6, SNRPD1, SNX4, NOL11, EDEM1, MAN2A1, COX4NB, TRAPPC2, SMARCE1, SEH1L, SRP72, TRNT1, RPL9, GPRC5A, DSTN, AP1S2, SERPINB4, MCFD2, TTRAP, GNAI3, CBFB, HSPE1, TMED10, OGT, MRPS28, MRPL1, FBXL3, HDAC2, NXT2, NDUFV2, HMGB1, TATDN3, POP4, IGF2BP3, ANXA10, EIF2S2, COPS3, MSH2, CASP1, PPA1, CAV2, TMED2, SERBP1, ASF1A, TRAK2, SMS, RPL26, GTF2H3, MMD, ELL2, TEX10, SF3B14, RDH10, ERO1L, VEZT, SNAP25, CAPZA2, FRG1, ANGPTL4, HMGN2, ADSS, PTPRF, SRP9, NT5C2, HIF1A, RBMX, GNPAT, C2orf3, UMPS, MAL2, FAM5C, SMARCA5, MRPL3, PLA2G4A, ASAM, SPRED1, KARS, SCAMP1, DUSP6, BZW1, ACTR3, FTH1, PLEK2, PHC2, TDG, AHCTF1, ASNS, CD164, ABCD3, UCHL5, SOCS6, ACSL4, RPL26L1, PERP, C1D, MTIF2, XPOT, MAGI3, ZMPSTE24, SDHD, RPS7, RP6-213H19.1, LAMB1, PPP1CB, GRB10, ANXA2, TAF9, UBE2N, INSIG2, DEPDC1, DMD, UTP14A, SLC25A12, RPE, RBL2, EEF1A1, TMED5, PHTF2, CLIC4, ADD3, CTSC, LYPLA1, WWP1, XRN2, ERRFI1, CSDA, ITGA1, MPHOSPH6, PRPF40A, CTNNAL1, POLK, MTMR12, TIMP3, EIF2S3, PGK1, PRDX3, TPRKB, ORC5L, GLRX, PSME3, USP16, ST13, PPIL1,	266

		EXT1, MTRR, PRNP, CYP51A1, AASDHPPT, YIPF4, LMAN2L, TMSL2, ARNT2, ACTR6, FAIM3, ZNF195, SKIV2L2, STAU2, CAV1, LIPA, NTS, ODC1, TAF9B, ATP5F1, PI4K2B, SMN2, TRMT5, ACTB, GNL3L, ZC3H8, RPL5, MYO5C, SUB1, TSNAX, DAZAP1, LSM5, CAPZA1, NARG2, RFX5, UBE3C, SC5DL, NT5E, CYP26B1, IMPA1, CREG1, SH2D2A, RPL6, PSMA1, UFM1, CYB5B, EIF4G2, NAP1L1, SYNCRIP, BTBD10, PNPT1, CCNE2, NDUFC2, TOMM20, SQRDL, PSMA2, ACTG2, PHLDA1, PAIP1, EEF1B2, MRPL50, PSMA6, IARS, CAP1, CBR4, PRKRIR, APIP, EGLN3, LMAN1, F2R, PRMT3, IGFBP3, SOAT1, OSTM1, YWHAQ, MTMR6, GLO1, MRPS35, C14orf166, LXN, UBE2V2, ITGA2, DARS2, EEF1E1, PPIL3, ANXA3, CDC40, MRPL42, SPCS2, CNIH, SCYL2, VPS26A, CDR2, ADH5, TMSL3, FAM82B, CD44, RIN2, COPS2, SMARCAD1, GCLC, GGH, TMEM50A, FNDC3A, RNF13, POLR3G, RPS3A, TMEM38B, NEIL3, ZNF277, SFRS3, CACYBP, NDFIP1, PTGES, GCSH	
Cellular component	Macromolecular complex	NDUFC2, TOMM20, GNG11, PSMA2, ACTR3, NPM1, PPIL1, FTH1, MOCS2, EEF1B2, MRPL50, PSMA6, AHCTF1, SNRPD1, KCTD16, HIGD1A, UCHL5, ARNT2, RPL26L1, SMARCE1, SEH1L, IGFBP3, SRP72, C1D, RPL9, SKIV2L2, STAU2, AP1S2, YWHAQ, MRPS35, CAV1, XPOT, ATP5F1, TAF9B, SMN2, UBE2V2, RPS7, ITGA2, LAMB1, OGT, PPP1CB, ACTB, MRPS28, ANXA2, MRPL1, TAF9, FBXL3, PPIL3, UBE2N, HDAC2, NDUFV2, CDC40, INSIG2, MRPL42, DMD, UTP14A, SPCS2, POP4, TGFBR2, RBL2, RPL5, EEF1A1, VPS26A, MYO5C, SUB1, EIF2S2, CLIC4, LSM5, KCNN4, COPS3, CAPZA1, MSH2, FAM82B, WWP1, CD44, UBE3C, ITGA1, ULBP2, CAV2, ASF1A, GTF2H3, RPL26, COPS2, CREG1, ELL2, GCLC, RPL6, PSMA1, CAPZA2, SNAP25, FRG1, EIF4G2, RPS3A, POLR3G, SRP9, NAP1L1, SYNCRIP, HIF1A, PRDX3, RBMX, ORC5L, SMARCA5, PSME3, MRPL3, GCSH	105
Cellular component	Intracellular	PTP4A1, TMED7, ACTG1, EIF1AX, NPM1, GLUD1, NT5C3, MOCS2, SNX6, SNRPD1, SNX4, NOL11, EDEM1, MAN2A1, COX4NB, TRAPPC2, SMARCE1, SEH1L, SRP72, TRNT1, RPL9, GPRC5A, DSTN, AP1S2, ZNF673, SERPINB4, MCFD2, TTRAP, GNAI3, CBFB, HSPE1,	272

		TMED10, OGT, MRPS28, MRPL1, FBXL3, HDAC2, NXT2, NDUFV2, HMGB1, TATDN3, POP4, IGF2BP3, ANXA10, EIF2S2, COPS3, MSH2, CASP1, PPA1, CAV2, TMED2, SERBP1, ASF1A, TRAK2, SMS, RPL26, GTF2H3, MMD, ELL2, TEX10, SF3B14, RDH10, ERO1L, VEZT, SNAP25, CAPZA2, FRG1, ANGPTL4, HMGN2, ADSS, PTPRF, SRP9, NT5C2, HIF1A, RBMX, GNPAT, C2orf3, C6orf62, UMPS, MAL2, FAM5C, SMARCA5, MRPL3, PLA2G4A, ASAM, SPRED1, KARS, SCAMP1, DUSP6, BZW1, ACTR3, FTH1, PLEK2, PHC2, TDG, AHCTF1, ASNS, CD164, ABCD3, UCHL5, SOCS6, ACSL4, RPL26L1, PERP, C1D, MTIF2, XPOT, MAGI3, ZMPSTE24, SDHD, RPS7, RP6-213H19.1, LAMB1, PPP1CB, GRB10, ANXA2, TAF9, UBE2N, INSIG2, DEPDC1, DMD, UTP14A, SLC25A12, RPE, RBL2, EEF1A1, ADD3, CLIC4, TMED5, PHTF2, CTSC, LYPLA1, WWP1, XRN2, ERRFI1, CSDA, ITGA1,MPHOSPH6, PRPF40A, DEPDC1B, CTNNAL1, POLK, MTMR12, TIMP3, EIF2S3, PGK1, PRDX3, TPRKB, ORC5L, RNF138, GLRX, PSME3, USP16, ST13, PPIL1, EXT1, MTRR, PRNP, CYP51A1, AASDHPPT, YIPF4, LMAN2L, TMSL2, ARNT2, ACTR6, FAIM3, ZNF195, SKIV2L2, STAU2, GMFB, CAV1, LIPA, NTS, ODC1, TAF9B, ATP5F1, PI4K2B, SMN2, TRMT5, ACTB, GNL3L, ZC3H8, RPL5, MYO5C, SUB1, TSNAX, DAZAP1, LSM5, CAPZA1, NARG2, RFX5, UBE3C, SC5DL, NT5E, CYP26B1, IMPA1, CREG1, SH2D2A, RPL6, PSMA1, UFM1, CYB5B, EIF4G2, BCAR3, NAP1L1, SYNCRIP, BTBD10, PNPT1, CCNE2, NDUFC2, TOMM20, SQRDL, PSMA2, ACTG2, PHLDA1, PAIP1, EEF1B2, MRPL50, PSMA6, IARS, CAP1, CBR4, PRKRIR, APIP, EGLN3, LMAN1, F2R, PRMT3, IGFBP3, SOAT1, OSTM1, YWHAQ, MTMR6, GLO1, MRPS35, C14orf166, LXN, UBE2V2, ITGA2, DARS2, EEF1E1, PPIL3, ANXA3, CDC40, MRPL42, SPCS2, CNIH, SCYL2, VPS26A, CDR2, ADH5, TMSL3, FAM82B, CD44, RIN2, COPS2, SMARCAD1, GCLC, GGH, TMEM50A, FNDC3A, RNF13, RPS3A, POLR3G, TMEM38B, NEIL3, ZNF277, SFRS3, CACYBP, NDFIP1, PTGES, GCSH	
Cellular component	Ribonucleoprotein complex	SRP72, RPL26, CDC40, RPL9, MRPL42, SKIV2L2, UTP14A, RPL6, PSMA1, NPM1, MRPS35, PPIL1, POP4, FRG1, RPL5, RPS3A, MRPL50,	31

		SRP9, SNRPD1, SYNCRIP, LSM5, SMN2, RPS7, RBMX, ACTB, MRPS28, MRPL1, RPL26L1, TAF9, PPIL3, MRPL3	
Cellular component	Cytoplasmic part	PTP4A1, TMED7, ACTG1, EIF1AX, NPM1, GLUD1, EXT1, NT5C3, MOCS2, PRNP, MTRR, CYP51A1, AASDHPPT, SNRPD1, YIPF4, EDEM1, LMAN2L, MAN2A1, COX4NB, TRAPPC2, SRP72, TRNT1, RPL9, GPRC5A, STAU2, DSTN, AP1S2, CAV1, MCFD2, ODC1, NTS, LIPA, GNAI3, HSPE1, ATP5F1, SMN2, TMED10, OGT, ACTB, MRPS28, MRPL1, NDUFV2, RPL5, ANXA10, TSNAX, EIF2S2, SC5DL, PPA1, CAV2, TMED2, SERBP1, SMS, CYP26B1, RPL26, MMD, RDH10, ERO1L, RPL6, VEZT, SNAP25 CAPZA2, EIF4G2, CYB5B, SRP9, PTPRF, NT5C2, NAP1L1, SYNCRIP, GNPAT, MAL2, PNPT1, MRPL3, PLA2G4A, CCNE2, KARS, NDUFC2, TOMM20, SCAMP1, SQRDL, ACTR3, FTH1, PHLDA1, EEF1B2, MRPL50, ASNS, CD164, PSMA6, IARS, CAP1, ABCD3, CBR4, ACSL4, RPL26L1, LMAN1, F2R, PERP, SOAT1, MTIF2, OSTM1, MRPS35, C14orf166, ZMPSTE24, SDHD, RPS7, RP6-213H19.1, LAMB1, ITGA2, DARS2, PPP1CB,ANXA2, EEF1E1, INSIG2, DMD, MRPL42, SLC25A12, SPCS2, CNIH, RPE, SCYL2, EEF1A1, VPS26A, ADH5, TMED5, PHTF2, CLIC4, ADD3, CTSC, LYPLA1, CD44, ITGA1, GCLC, GGH, TMEM50A, FNDC3A, RPS3A, TMEM38B, PRDX3, EIF2S3, TPRKB, GLRX, NDFIP1, GCSH	142
Cellular component	Cell fraction	ASAM, PLA2G4A, KARS, DMD, DUSP6, ACTG1, SPCS2, ADH5, CYP51A1, CLIC4, ASNS, IARS, SLC16A6, ACSL4, LMAN1, ITGA1, CAV2, TMED2, NT5E, SOAT1, CYP26B1, FOLR3, CD55, MMD, SH2D2A, CYSLTR1, RDH10, ERO1L, YWHAQ, GLO1, SNAP25, CAV1, FNDC3A, LIN7C, CYB5B, GNAI3, NTS, ZMPSTE24, PTPRF, SYNCRIP, TMED10, RYK, SLC16A1, ACTB, ANXA2, PTGES	46
Cellular component	Zymogen granule	PLA2G4A, SCAMP1, TMED10, GNAI3, TMED2	5
Cellular component	Nuclear part	CCNE2, HMGB1, CDC40, DUSP6, UTP14A, NPM1, PPIL1, POP4, PHLDA1, RBL2, RPL5, SUB1, DAZAP1, ADD3, AHCTF1, TDG, SNRPD1, NOL11, COPS3, PRKRIR, MSH2, XRN2, ARNT2, SMARCE1, MPHOSPH6, SEH1L, ASF1A, PRPF40A, GTF2H3, RPL9, C1D, COPS2,	63

		CREG1, SKIV2L2, ELL2, SMARCAD1, STAU2, TEX10, MTMR6, FRG1, TTRAP, XPOT, RPS3A, POLR3G, TAF9B, NAP1L1, TMEM38B, SYNCRIP, SMN2, HIF1A, RPS7, RBMX, ORC5L, OGT, PPP1CB, GNL3L, ACTB, CACYBP, SMARCA5, TAF9, PTGES, PPIL3, HDAC2	
Cellular component	Vesicular fraction	SOAT1, CYP26B1, CYP51A1, PTPRF, SYNCRIP, DMD, TMED10, ERO1L, RDH10, SPCS2, ACSL4, FNDC3A, LMAN1, CYB5B, PTGES, TMED2	16
