# Supplementary material for: Cancer-initiating cells derived from established cervical cell lines exhibit stem-cell markers and increased radioresistance
Source: BMC Cancer. 2012 Jan 28;12:48. doi: 10.1186/1471-2407-12-48 (PMC3299592; doi:10.1186/1471-2407-12-48)
Supplement: Additional file 5 — Table S4- Genes. Biological functions of the genes with altered down-regulated expression by a factor of at least 1.5-fold in HeLa spheroid cells compared with HeLa monolayer cells, as determined by WebGestalt (Gene Set Analysis Toolkit). [file 1471-2407-12-48-S5.PDF]

Supplementary Table 4. Biological functions of the genes with altered down-regulated expression by a factor of at least 1.5-fold in HeLa spheroid cells compared with HeLa monolayer cells, as determined by WebGestalt (Gene Set Analysis Toolkit).

Category	Function	Gene Symbol	No. of molecules
Biological process	Response to protein stimulus	NR3C1, CYR61, ID2, DNAJB1, DDIT3, HSPB3, HERPUD1, HSPA6, HSPA1B	9
Biological process	Response to unfolded protein	DNAJB1, DDIT3, HERPUD1, HSPB3, HSPA1B, HSPA6	6
Biological process	Response to stress	C3, KCNIP3, OLR1, BTG2, KLRC3, EDN1, GRHL3, TP53, HIST2H2BE, C1R, DNAJB1, CXCL2, HERPUD1, HSPB3, HSPA1B, HSPA6, CTGF, TXNIP, GADD45B, ENO3, SREBF1, DDIT3, KLRK1, PPBP	24
Biological process	Response to chemical stimulus	OLR1, NR3C1, BTG2, MGP, EDN1, TP53, ID2, CXCL2, DNAJB1, GH1, CCNE1, HERPUD1, HSPB3, HSPA1B, HSPA6, CYR61, TXNIP, ENO3, DDIT3, PPBP	20
Biological process	Response to external stimulus	C3, OLR1, CTGF, CYR61, BTG2, MGP, ENO3, TXNIP, EDN1, SREBF1, GRHL3, TP53, C1R, CXCL2, CCNE1, DDIT3, PPBP	17
Biological process	Response to organic substance	NR3C1, CYR61, BTG2, MGP, TXNIP, ID2, DNAJB1, GH1, CCNE1, DDIT3, HERPUD1, HSPB3, HSPA1B, HSPA6	14
Biological process	Response to stimulus	OLR1, KLRC3, MGP, TP53, ID2, HIST2H2BE, DNAJB1, CXCL2, GH1, CCNE1, HSPB3, CTGF, CYR61, ENO3, TXNIP, SREBF1, KLRK1, PPBP, C3, CRB1, KCNIP3, NR3C1, BTG2, GRHL3, EDN1, TNFSF10, C1R, OR4X1, HERPUD1, HSPA6, HSPA1B, GADD45B, DDIT3	33
Biological process	Response to biotic stimulus	TP53, HIST2H2BE, DNAJB1, DDIT3, HSPB3, HERPUD1, HSPA6, PPBP, HSPA1B	9
Biological process	Cellular response to biotic stimulus	DDIT3, HERPUD1, TP53	3
Biological process	ER-nuclear signaling pathway	DDIT3, HERPUD1, TP53	3

Molecular function	Extracellular matrix binding	OLFML2A, DCN, CYR61	3
Molecular function	Receptor binding	C3, SPRED2, TRIP12, CTGF, EDN1, TNFSF10, CXCL2, GH1, CCNE1, INSL4, KLRK1, PPBP	12
Molecular function	Carbohydrate binding	DCN, OLR1, CTGF, PFKL, CYR61, KLRC3, KLRK1	7
Molecular function	Cytokine receptor binding	TNFSF10, SPRED2, CXCL2, GH1, PPBP	5
Molecular function	Transcription repressor activity	ID2, HES1, DMAP1, HESX1, KCNIP3, DDIT3	6
Molecular function	Protein N-terminus binding	DCN, HESX1, TP53	3
Molecular function	Insulin-like growth factor binding	CTGF, CYR61	2
Molecular function	Hormone receptor binding	TRIP12, GH1, CCNE1	3
Molecular function	Transcription factor binding	DMAP1, KCNIP3, TRIP12, CCNE1, DDIT3, RFXANK, TP53	7
Molecular function	Calcium-dependent protein binding	KCNIP3, MGP	2
Cellular component	Extracellular region	CRB1, C3, OLFML2A, DCN, COL9A3, OLR1, MGP, TMPRSS2, EDN1, TNFSF10, C1R, CXCL2, GH1, INSL4, CTRB1, CTGF, CYR61, SERPINI1, CFHR4, LUZP2, PPBP	21
Cellular component	Extracellular region part	CTRB1, C3, OLFML2A, DCN, COL9A3, CTGF, MGP, EDN1, TNFSF10, CXCL2, GH1, INSL4, PPBP	13
Cellular component	Extracellular space	CTRB1, C3, MGP, EDN1, TNFSF10, CXCL2, GH1, INSL4, PPBP	9
Cellular component	Replication fork	DMAP1, TP53	2
Cellular component	Cytosol	ABL2, MCM3AP, IDI2, KCNIP3, RPL28, SLC12A3, PFKL, ENO3, TP53, ID2, CCNE1	11
Cellular component	Extracellular	OLFML2A, DCN, COL9A3, CTGF, MGP	5

	matrix		
Cellular component	Cytosolic part	RPL28, PFKL, ENO3	3
Cellular component	Vesicle	CAMKV, SPRED2, SLC12A3, SYNGR3, PPBP, SREBF1, GPR120	7
Cellular component	Chromosomal part	ID2, HIST2H2BE, DMAP1, HIST1H2AD, TP53	5
Cellular component	Large ribosomal subunit	RPL28, MRPL13	2
