# Supplementary material for: Cancer-initiating cells derived from established cervical cell lines exhibit stem-cell markers and increased radioresistance
Source: BMC Cancer. 2012 Jan 28;12:48. doi: 10.1186/1471-2407-12-48 (PMC3299592; doi:10.1186/1471-2407-12-48)
Supplement: Additional file 6 — Table S5- Genes. Biological functions of the genes with altered up-regulated expression by a factor of at least 1.5-fold in SiHa spheroid cells compared with SiHa monolayer cells, as determined by WebGestalt (Gene Set Analysis Toolkit). [file 1471-2407-12-48-S6.PDF]

Supplementary Table 5. Biological functions of the genes with altered up-regulated expression by a factor of at least 1.5-fold in SiHa spheroid cells compared with SiHa monolayer cells, as determined by WebGestalt (Gene Set Analysis Toolkit).

**Category**	**Function**	**Gene Symbol**	**No. of molecules**
Biological process	Translational elongation	RPL3, RPS20, RPS2, RPS29, RPL30, RPS5, EEF1A1, RPL5, RPL13A, RPL18, RPS11, RPL39, RPL27, RPS6, RPL19, RPS21, RPS18, RPS23, RPS13, RPL26, RPL9, RPL35, RPL31, RPL41, RPS10, RPL7,RPS27, RPL18A, RPL27A, RPLP0, RPS3A, SRP9, RPL7A, RPL35A, RPS15A, RPS7, RPS12, RPS3	38
Biological process	Translation	RPL3, RPS20, RPS2, MRPS2, RPS29, RPL30, RPS5, EEF1A1, RPL5, RPL13A, RPL18, RPS11, RPL39, EIF1, RPL27, RPS6, RPL19, RPS21, RPS18, RPL22L1, RPS23, RPS13, RPL26, RPL9, RPL35, RPL31, RPL41, RPL7, RPS10, EIF4A1, RPS27, RPL18A, RPL27A, RPLP0, RPS3A, RPL7A, SRP9, RPL35A, RPS15A, RPS7, IL6, PPP1R15A, EIF4EBP1, RPS12, RPS3	45
Biological process	Cellular protein metabolic process	DUSP6, RPS29, LDLR, RPL30, RPS5, PPP2R2A, TAF6L, CSTA, CCT4, RPL18, PSMD14, RPS6, RPS21, PPP1CA, TGM2, RPS18, PDIA6, TIPARP, AGA, RPS13, LOX, LIF, RPL35, RPL9, HSP90AB1, RPL31, CDC20, RPL18A, RPS27, HSPE1, RPL35A, TCP1, PSMD13, UBE2V2, RPS7, EIF4EBP1, CCT6A, USP11, TAF9, RPS3, UBE2N, RPS20, RPL3, RPS2, HSP90B1, MRPS2, PMVK, RPL5, EEF1A1, RPL13A, DNAJB9, BMP2, TIMP1, SDCCAG10, RPS11, RPL39, EIF1, RPL27, WWP1, RPL19, MAPK11, RPL22L1, WNK2, RPS23, DUSP1, VEGFC, RPL26, TNFAIP3, PLAUR, ERO1L, RPL41, RPL7, RPS10, EIF4A1, RPL27A, RPLP0, CCT8, RPS3A, RPL7A, SRP9, TRIB3, RPS15A, IL6, PPP1R15A, RPS12	85
Biological process	Protein metabolic process	DUSP6, RPS29, LDLR, RPL30, RPS5, PPP2R2A, TAF6L, CSTA, CCT4, RPL18, PSMD14, RPS6, RPS21, PPP1CA, TGM2, TINAGL1, RPS18, PDIA6, TIPARP, AGA, RPS13, LOX, ATP6AP2, LIF, RPL35, CD55, RPL9, HSP90AB1, RPL31, CDC20, RPL18A, RPS27, HSPE1, RPL35A,	92

		TCP1, PSMD13, UBE2V2, RPS7, EIF4EBP1, CCT6A, USP11, TAF9, RPS3, UBE2N, RPS20, RPL3, HSP90B1, RPS2, SERPINE2, MRPS2, PMVK, EEF1A1, RPL5, DNAJB9, RPL13A, BMP2, TIMP1, SDCCAG10, CTSC, RPS11, RPL39, EIF1, RPL27, WWP1, RPL19, MAPK11, CTSD, CASP1, RPL22L1, WNK2, RPS23, DUSP1, VEGFC, RPL26, TNFAIP3, PLAUR, ERO1L, RPL41, RPL7, RPS10, EIF4A1, RPL27A, RPLP0, CCT8, RPS3A, RPL7A, SRP9, TRIB3, RPS15A, IL6, PPP1R15A, RPS12	
Biological process	Apoptosis	HSP90B1, TUBB2C, NPM1, IL1A, BCAP31, SERPINB2, SH3KBP1, PRNP, TNFRSF6B, PDCD10, ASNS, NEFL, RPS6, TNFRSF12A, TGM2, CASP1, DUSP1, TNFAIP3, BNIP3L, LGALS1, MCL1, ADRB2, ANGPTL4, HSPE1, ADM, RPS3A, TRIB3, BTG1, PTGS2, GADD45A, PRDX3, IL6, PPP1R15A, TNFRSF10D, RPS3, TAF9, FOSL1, TPT1	38
Biological process	Ribosomal large subunit biogenesis	NPM1, RPL26, RPL35A, RPL5, RPL7	5
Biological process	Macromolecule metabolic process	ZNF426, DUSP6, RPS29, LDLR, RPL30, RPS5, PPP2R2A, NPM1, TAF6L, ISOC2, IL1A, CSTA, CCT4, RPL18, LDHC, FOSB, PSMD14, RPS6, RPS21, TGM2, PPP1CA, TINAGL1, RPS18, PDIA6, ENO1, TIPARP, AGA, RPS13, LOX, ATP6AP2, LIF, RPL35, CD55, RPL9, HSP90AB1, RPL31, CDC20, BNIP3L, YWHAQ, RPL18A, RPS27, RFC2, CDCA7, HSPE1, RPL35A, TCP1, NUDT21, PSMD13, UBE2V2, RPS7, EIF4EBP1, XRCC6, CT6A, USP11, TAF9, RPS3, FOSL1, UBE2N, RPL3, RPS20, HSP90B1, RPS2, EGR1, SERPINE2, MRPS2, RASD1, CRABP2, PMVK, EEF1A1, RPL5, DNAJB9, RPL13A, BMP2, SUB1, TIMP1, SDCCAG10, CTSC, RPS11, RPL39, EIF1, RPL27, WWP1, RPL19, MAPK11, CTSD, CASP1, RPL22L1, RBMS2, WNK2, RPS23, DUSP1, LDHB, VEGFC, RPL26, TK1, TNFAIP3, COPS2, RALY, RTEL1, PLAUR, ERO1L, RPL41, RPL7, RPS10, EIF4A1, ADRB2, RPL27A, FOS, RPLP0, CCT8, RPS3A, RQCD1, RPL7A, SRP9, TRIB3, BTG1, GADD45A, RPS15A, PRDX3, POLR1D, IL6, PPP1R15A, ORC5L, LRPPRC, RPS12, GALE	126
Biological process	Metabolic process	LDLR, NPM1, TAF6L, ISOC2, IL1A, PRNP, CCT4, RPL18, FOSB, RPS6, ETFA, RPS21, TGM2, PPP1CA, TINAGL1, RPS18, TIPARP, ENO1, AGA, ATP6AP2, LIF, CD55, RPL9, RPL31, BNIP3L, FABP5, ODC1,	152

		RFC2, CDCA7, HSPE1, ATP5F1, RPL35A, NUDT21, PSMD13, CYB561D2, CCT6A, UQCRH, RPS3, FOSL1, EGR1, SERPINE2, MRPS2, RPL5, RPL13A, BMP2, SUB1, TIMP1, SC4MOL, RPS11, RPL39, CTSD, CASP1, WNK2, RPS23, SMS, FDFT1, SUCLA2, RPL26, RALY, ERO1L, RPS10, ADRB2, NPC2, CCT8, RPL7A, SRP9, MTHFD2, POLR1D, PPP1R15A, ACADVL, GALE, ZNF426, DUSP6, RPS29, UCK2, PPP2R2A, RPS5, RPL30, FTH1, CSTA, ASNS, LDHC, PSMD14, PDIA6, LOX, RPS13, RPL35, HSP90AB1, CDC20, YWHAQ, RPS27, RPL18A, ADM, SDHD, EDN2, PTGS2, TCP1, RPS7, UBE2V2, EIF4EBP1, XRCC6, P4HA1, TAF9, USP11, UBE2N, RPS20, RPL3, RPS2, HSP90B1, RASD1, CRABP2, PMVK, EEF1A1, DNAJB9, CTSC, SDCCAG10, EIF1, RPL27, LYPLA1, MAPK11, RPL19, SHMT2, WWP1, RPL22L1, RBMS2, HSD17B12, LDHB, DUSP1, VEGFC, TK1, TNFAIP3, COPS2, RTEL1, PLAUR, RPL41, RPL7, EIF4A1, GGH, RPL27A, FOS, RPLP0, RPS3A, RQCD1, TRIB3, BTG1, GADD45A, RPS15A, PRDX3, IL6, ORC5L, LRPPRC, RPS12	
Biological process	Cellular metabolic process	LDLR, NPM1, TAF6L, IL1A, CCT4, RPL18, FOSB, RPS6, ETFA, RPS21, TGM2, PPP1CA, RPS18, ENO1, TIPARP, AGA, LIF, RPL9, RPL31, ODC1, RFC2, CDCA7, HSPE1, ATP5F1, RPL35A, NUDT21, PSMD13, CYB561D2, CCT6A, UQCRH, RPS3, FOSL1, EGR1, MRPS2, RPL5, RPL13A, BMP2, SUB1, TIMP1, SC4MOL, RPS11, RPL39, CTSD, WNK2, RPS23, SMS, FDFT1, SUCLA2, RPL26, RALY, ERO1L, RPS10, ADRB2, NPC2, CCT8, RPL7A, SRP9, MTHFD2, POLR1D, PPP1R15A, ACADVL, GALE, ZNF426, DUSP6, RPS29, PPP2R2A, RPS5, RPL30, CSTA, ASNS, LDHC, PSMD14, PDIA6, LOX, RPS13, RPL35, HSP90AB1, CDC20, YWHAQ, RPS27, RPL18A, ADM, SDHD, EDN2, PTGS2, TCP1, RPS7, UBE2V2, EIF4EBP1, XRCC6, TAF9, USP11, UBE2N, RPS20, RPL3, RPS2, HSP90B1, RASD1, CRABP2, PMVK, EEF1A1, DNAJB9, SDCCAG10, EIF1, RPL27, LYPLA1, MAPK11, RPL19, SHMT2, WWP1, RPL22L1, RBMS2, HSD17B12, LDHB, DUSP1, VEGFC, TK1, COPS2, TNFAIP3, RTEL1, PLAUR, RPL7, RPL41, GGH, EIF4A1, FOS, RPL27A, RPLP0, RPS3A, RQCD1, TRIB3, BTG1, GADD45A, RPS15A, PRDX3, IL6, ORC5L, LRPPRC, RPS12	139

Molecular function	Structural constituent of ribosome	RPL3, RPS20, RPS2, MRPS2, RPS29, RPL30, RPS5, RPL5, RPL13A, RPL18, RPL39, RPS11, RPL27, RPS6, RPL19, RPS21, RPS18, RPL22L1, RPS23, RPS13, RPL26, RPL9, RPL35, RPL31, RPL7, RPL41, RPS27, RPL18A, RPL27A, RPLP0, RPS3A, RPL7A, RPL35A, RPS15A, RPS7, RPS12, RPS3	37
Molecular function	Structural molecule activity	RPL3, RPS20, RPS2, ACTG1, TUBB2C, MRPS2, RPS29, RPL30, RPS5, CSTA, RPL5, RPL13A, RPL18, RPL39, RPS11, NEFL, RPL27, RPS6, RPL19, RPS21, TINAGL1, RPS18, RPL22L1, RPS23, RPS13, RPL26, RPL9, RPL35, RPL31, RPL41, RPL7, RPS27, RPL18A, RPL27A, RPLP0, RPS3A, RPL7A, RPL35A, RPS15A, RPS7, RPS12, RPS3	42
Molecular function	RNA binding	RPL3, RPS20, HSP90B1, RPS2, RPL30, RPS5, NPM1, RPL5, RPL18, RPL39, RPS11, RPL19, RPS18, RBMS2, RPS13, SRP72, RPL26, RPL9, RPL35, RALY, RPL31, RPL7, RPL41, RPL18A, EIF4A1, RPL27A, RPLP0, RPS3A, SRP9, RPL7A, RPL35A, NUDT21, RPS15A, RPS7, LRPPRC, RPS3	36
Molecular function	Protein binding	ACTG1, LDLR, NPM1, RCHY1, TAF6L, ISOC2, IL1A, SH3KBP1, CCT4, PRNP, TNNT1, FOSB, RPS6, RPS21, PCOLCE2, TGM2, PPP1CA, TIPARP, ENO1, STC1, AGA, ATP6AP2, LIF, KLHL4, RPL31, BNIP3L, FABP5, MCL1, NUTF2, ODC1, RFC2, HSPE1, ATP5F1, RPL35A, NUDT21, PSMD13, LRRC40, IL8, SSR3, CCT6A, UQCRH, TNFRSF10D, RPS3, FOSL1, IL13RA2, CXCL3, RHOC, RPL5, C1QBP, TNFRSF6B, BMP2, SUB1, TIMP1, COPS3, RPS11, TNFRSF12A, SDCBP, CASP1, WNK2, FDFT1, SUCLA2, RPL26, ERO1L, RPS10, NPC2, CAPZA2, ADRB2, CCT8, ANGPTL4, SRP9, POLR1D, PPP1R15A, GALE, DUSP6, TUBB2C, RPS29, PPP2R2A, FTH1, CSTA, BCAP31, ASNS, PSMD14, ITGB6, PDIA6, LOX, RPS13, RPL35, HSP90AB1, CDC20, NARF, LGALS1, YWHAQ, RPS27, RPL18A, ADM, S100A16, EDN2, PTGS2, TCP1, RPS7, UBE2V2, EIF4EBP1, XRCC6, CXCL1, TAF9, USP11, UBE2N, THBD, RPS20, RPL3, RPS2, HSP90B1, IGFBP6, RASD1, CXCL2, PMVK, EEF1A1, DNAJB9, PDCD10, CTSC, TMSB10, NEFL, MAPK11, SHMT2, WWP1, ERRFI1, HSD17B12, S100A6, DUSP1, LDHB, VEGFC, TNFAIP3, COPS2, CCL20, PLAUR, RPL7, STMN3, EIF4A1, FOS, RPLP0, DKK1, RPS3A, RQCD1, TRIB3,	153

		BTG1, GADD45A, RPS15A, PRDX3, IL6, ORC5L, LRPPRC, SERPINB8, TPT1	
Molecular function	Unfolded protein binding	HSP90B1, TCP1, TUBB2C, HSP90AB1, NPM1, CCT6A, CCT8, DNAJB9, HSPE1, CCT4	10
Molecular function	Binding	ACTG1, LDLR, NPM1, RCHY1, TAF6L, ISOC2, FABP5L3, IL1A, SH3KBP1, CCT4, PRNP, RPL18, TNNT1, FOSB, RPS6, ETFA, RPS21, PCOLCE2, TINAGL1, PPP1CA, TGM2, RPS18, TIPARP, ENO1, STC1, AGA, ATP6AP2, LIF, SRP72, RPL9, KLHL4, RPL31, BNIP3L, SUMF2, MCL1, FABP5, NUTF2, RFC2, ODC1, HSPE1, ATP5F1, RPL35A, NUDT21, PSMD13, LRRC40, CYB561D2, IL8, SSR3, CCT6A, UQCRH, TNFRSF10D, RPS3, FOSL1, IL13RA2, EGR1, SERPINE2, CXCL3, RHOC, RPL5, C1QBP, ANXA10, TNFRSF6B, BMP2, SUB1, TIMP1, SC4MOL, RPS11, RPL39, COPS3, TNFRSF12A, SDCBP, CASP1, WNK2, FDFT1, SUCLA2, RPL26, RALY, ERO1L, RPS10, NPC2, CAPZA2, ADRB2, CCT8, ANGPTL4, SRP9, RPL7A, MTHFD2, POLR1D, PPP1R15A, ACADVL, GALE, ZNF426, DUSP6, TUBB2C, RPS29, UCK2, PPP2R2A, RPS5, RPL30, FTH1, CSTA, BCAP31, ASNS, LDHC, PSMD14, ITGB6, PDIA6, LOX, RPS13, RPL35, HSP90AB1, SLC39A8, CDC20, NARF, LGALS1, YWHAQ, RPS27, RPL18A, ADM, S100A16, SDHD, EDN2, PTGS2, TCP1, RPS7, UBE2V2, EIF4EBP1, XRCC6, P4HA1, CXCL1, USP11, TAF9, UBE2N, THBD, RPL3, RPS20, ANXA3, HSP90B1, RPS2, IGFBP6, RASD1, CXCL2, CRABP2, PMVK, EEF1A1, DNAJB9, PDCD10, TMSB10, CTSC, EIF1, NEFL, WWP1, SHMT2, RPL19, MAPK11, ERRFI1, S100A6, HSD17B12, RBMS2, DUSP1, LDHB, VEGFC, TK1, TNFAIP3, COPS2, RTEL1, CCL20, PLAUR, RPL41, RPL7, STMN3, EIF4A1, RPL27A, FOS, RPLP0, DKK1, RPS3A, RQCD1, TRIB3, BTG1, GADD45A, RPS15A, PRDX3, RND3, IL6, ORC5L, LRPPRC, KCNK4, SERPINB8, TPT1	90
Molecular function	rRNA binding	NPM1, RPL9, RPS18, RPS11, RPL5	5
Molecular function	mRNA binding	RPS5, EIF4A1, RPL35, RPS3, RPL7, RPS13	6

Molecular function	Cytokine receptor binding	VEGFC, LIF, CCL20, IL6, CXCL3, IL8, CXCL1, SDCBP, CXCL2, IL1A	10
Molecular function	Chemokine activity	CXCL1, CXCL2, CCL20, CXCL3, IL8	5
Cellular component	Cytosolic ribosome	RPL3, RPS20, RPL26, RPS2, RPL35, RPL31, RPS29, RPL41, RPL7, RPS10, RPL30, RPS5, RPL18A, RPS27, RPL27A, RPLP0, RPL5, RPS3A, RPL7A, RPL18, RPS15A, RPS7, RPL39, RPS11, RPS6, RPL19, RPS21, RPS12, RPS3, RPS18, RPS23, RPS13	32
Cellular component	Cytosolic part	RPL3, RPS20, RPS2, RPS29, RPL30, RPS5, RPL5, CCT4, RPL18, RPL39, RPS11, NEFL, RPS6, RPL19, RPS21, RPS18, ENO1, RPS23, RPS13, RPL26, RPL35, RPL31, RPS10, RPL7, RPL41, RPS27, RPL18A, RPL27A, RPLP0, RPS3A, RPL7A, TCP1, RPS15A, PRDX3, RPS7, CCT6A, RPS12, RPS3	38
Cellular component	Ribosomal subunit	RPL3, RPS20, RPS2, MRPS2, RPS29, RPL30, RPS5, NPM1, RPL5, RPL13A, RPL18, RPL39, RPS11, RPS6, RPL19, RPS21, RPS18, RPS23, RPS13, RPL26, RPL35, RPL31, RPS10, RPL7, RPL41, RPS27, RPL18A, RPL27A, RPLP0, RPS3A, RPL7A, RPS15A, RPS7, RPS12, RPS3	35
Cellular component	Ribosome	RPL3, RPS20, RPS2, MRPS2, RPS29, RPL30, RPS5, NPM1, RPL5, RPL13A, MRPL50, RPL18, RPL39, RPS11, RPL27, RPS6, RPL19, RPS21, RPS18, RPL22L1, RPS23, RPS13, RPL26, RPL9, RPL35, RPL31, RPL41, RPL7, RPS10, RPS27, RPL18A, RPL27A, RPLP0, RPS3A, RPL7A, RPL35A, RPS15A, RPS7, RPS12, RPS3	40
Cellular component	Cytosol	ACTG1, TUBB2C, RPS29, UCK2, RPL30, RPS5, NPM1, FTH1, BCAP31, SH3KBP1, CCT4, ASNS, RPL18, RPS6, RPS21, PPP1CA, RPS18, ENO1, RPS13, RPL35, RPL9, RPL31, CDC20, RPL18A, RPS27, NUTF2, ODC1, RPL35A, TCP1, RPS7, EIF4EBP1, CCT6A, RPS3, FOSL1, RPS20, RPL3, RPS2, HSP90B1, RPL5, EEF1A1, RPL13A, RPL39, RPS11, NEFL, RPL27, MAPK11, RPL19, SDCBP, RPS23, SMS, RPL26, TK1, RPS10, RPL7, RPL41, GGH, EIF4A1, RPL27A, CCT8, RPLP0, RPS3A, RPL7A, RPS15A, PRDX3, RPS12, SERPINB8, GALE	67
Cellular component	Ribonucleoprotein complex	RPL3, RPS20, RPS2, MRPS2, RPS29, RPL30, RPS5, NPM1, RPL5, RPL13A, MRPL50, RPL18, RPL39, RPS11, RPL27, RPS6, RPL19, RPS21, RPS18, RPL22L1, RPS23, RPS13, SRP72, RPL26, RPL9, RPL35,	44

		RALY, RPL31, RPL41, RPL7, RPS10, RPS27, RPL18A, RPL27A, RPLP0, RPS3A, RPL7A, SRP9, RPL35A, RPS15A, RPS7, RPS12, RPS3, TAF9	
Cellular component	Cytosolic small ribosomal subunit	RPS20, RPS2, RPS15A, RPS7, RPS11, RPS29, RPS10, RPS6, RPS5, RPS27, RPS21, RPS12, RPS18, RPS3, RPS23, RPS3A, RPS13	17
Cellular component	Small ribosomal subunit	RPS20, RPS2, MRPS2, RPS29, RPS10, RPS5, NPM1, RPS27, RPS3A, RPS15A, RPS7, RPS11, RPS6, RPS21, RPS12, RPS3, RPS18, RPS23, RPS13	19
Cellular component	Cytosolic large ribosomal subunit	RPL3, RPL7A, RPL26, RPL35, RPL18, RPL39, RPL31, RPL41, RPL7, RPL30, RPL19, RPL18A, RPL27A, RPLP0, RPL5	15
Cellular component	Large ribosomal subunit	RPL3, RPL26, RPL35, RPL31, RPL41, RPL7, RPL30, NPM1, RPL18A, RPL27A, RPLP0, RPL5, RPL13A, RPL7A, RPL18, RPL39, RPL19	17
