# Supplementary material for: Cancer-initiating cells derived from established cervical cell lines exhibit stem-cell markers and increased radioresistance
Source: BMC Cancer. 2012 Jan 28;12:48. doi: 10.1186/1471-2407-12-48 (PMC3299592; doi:10.1186/1471-2407-12-48)
Supplement: Additional file 7 — Table S6- Genes. Biological functions of the genes with altered down-regulated expression by a factor of at least 1.5-fold in SiHa spheroid cells compared with SiHa monolayer cells, as determined by WebGestalt (Gene Set Analysis Toolkit). [file 1471-2407-12-48-S7.PDF]

Supplementary Table 6. Biological functions of the genes with altered down-regulated expression by a factor of at least 1.5-fold in SiHa spheroid cells compared with SiHa monolayer cells, as determined by WebGestalt (Gene Set Analysis Toolkit).

Category	Function	Gene Symbol	No. of molecules
Biological Process	Chromatin modification	SIRT1, DMAP1, TRRAP, SETD1B, SMARCAD1, SETD2, CBX2, CBX6, SUDS3, PCGF2, PRMT7, CHD4, SMARCD2, BRD1, SMARCA5, CARM1, CSRP2BP	17
Biological Process	Primary metabolic process	CYP24A1, CTSF, PRKAB2, RXRA, UGDH, SUDS3, CCNL2, SIN3A, DPP8, SPSB1, GLUL, CCNE1, SARS2, LBH, DMBX1, PAPSS1, MAN2A1, OGG1, CARM1, TULP4, C3, ZHX2, CELSR2, ZXDC, SETD1B, ASXL1, CBX6, ELAC2, BRD1, TYSND1, PLCXD2, MAN2A2, CD24, TRRAP, ZNF689, MKL2, OGT, DHTKD1, ST3GAL2, GAK, RUNX1, DUX1, B4GALT2, FUBP1, FABP3, UST, LRRFIP1, ATOH8, OLR1, ZHX3, TAF1C, RNF34, MAN2B1, PITPNM1, HOXC6, THBS1, MCM3AP, NFATC3, ENO3, PRKCI, ADCY6, IGF2BP1, UBE3C, MAPK7, TTLL1, ZNF562, CSRP2BP, ZNF629, ABCE1, CYP26B1, SELI, ABI2, DMAP1, SLC27A2, ASRGL1, SFRS14, SETD2, KIAA2018, DUSP9, C1R, CTSK, SLTM, LCAT, TSC1, FKBPL, SERTAD2, GGCX, CYP4F2, IGFBP5, PRMT7, CFB, SMARCA5, NCOA5, ZNF398, DMPK, USP7, ZNF207, HTRA1, GNG3, TP53, MYH9, MRPL30, LOC440897, MDC1, TNKS, MED4, IGFBP7, PUS3, SPTBN1, LMO4, NARFL, TXNIP, CBX2, CHD4, SOLH, OSBPL7, MGLL, GLYCTK, SIRT1, FIGNL1, ZNF500, PTPN9, ATF7IP2, TSEN54, STAT2, SOX12, OSBPL2, PEX5, DGCR8, PTK7, JRK, ADAT1, FOXF1, BMPR1A, ST3GAL1, SPHK2, PRDM2, ABCA1, MAPK12, SSH2, NCOR2, ATP9A, DIO2, AGPAT3, ZNF219, BMP6, PIP5K1C, HOXA10, CTRB1, POLR3D, SULT1A1, SLC25A10, MXD3, UBE3B, STAT3, FBXO17, CPS1, LONRF3, SMARCAD1, GCLC, TEF, RFXANK, FOXC1, CDK5RAP1, FEM1A, NEUROG3, NEIL3, TBX3, DEGS1, SLC7A5, PCGF2, CHST3, SMARCD2, PCYT1A, THOP1, MRPL23, TBCE	177

Biological process	Negative regulation of helicase activity	SIRT1, TP53	2
Biological process	Peptidyl-glutamic acid modification	GGCX, TTLL1	2
Biological process	Chromatin organization	SIRT1, DMAP1, TRRAP, SETD1B, SMARCAD1, SETD2, CBX2, CBX6, SUDS3, PCGF2, PRMT7, CHD4, SMARCD2, BRD1, SMARCA5, CARM1, CSRP2BP	17
Biological process	Positive regulation of inflammatory response to antigenic stimulus	C3, CD24	2
Biological process	Histone modification	SIRT1, DMAP1, TRRAP, SETD2, PCGF2, PRMT7, BRD1, CARM1, CSRP2BP	9
Biological process	Response to hormone stimulus	FABP3, CPS1, PHIP, GNG3, GCLC, RXRA, ASIP, CCNE1, THBS1, IGFBP7, LCAT, TSC1, CD24, TXNIP, PRKCI, ADCY6, STAT3	17
Biological process	Transcription from RNA polymerase II promoter	SIRT1, LRRFIP1, ZHX2, NCOR2, TEF, TAF1C, RXRA, TP53, SIN3A, BMP6, MED4, FOXC1, HOXC6, STAT2, SOX12, NFATC3, DMBX1, NEUROG3, LMO4, TBX3, MKL2, CBX2, PCGF2, CHD4, SMARCD2, RUNX1, SMARCA5, DUX1, STAT3, FUBP1	30
Biological process	Mannose metabolic process	MAN2A2, MAN2B1, MAN2A1	3
Molecular function	Mannosyl-oligosaccharide 1,3-1,6-alpha-mannosidase activity	MAN2A2, MAN2A1	2
Molecular function	Alpha-mannosidase activity	MAN2A2, MAN2B1, MAN2A1	3
Molecular function	Transcription regulator activity	ZNF207, LRRFIP1, NCOR2, ATOH8, ZHX3, TAF1C, RXRA, TP53, ZNF219, SIN3A, MED4, CCNE1, HOXC6, HOXA10, NFATC3, DMBX1,	48

		LMO4, CBX2, MXD3, SOLH, STAT3, CARM1, TULP4, SIRT1, ZHX2, DMAP1, ZXDC, ZNF500, TEF, KIAA2018, RFXANK, FOXC1, STAT2, SOX12, SERTAD2, TRRAP, NEUROG3, TBX3, MKL2, PCGF2, SMARCD2, FOXF1, RUNX1, SMARCA5, DUX1, PRDM2, ZNF398, FUBP1	
Molecular function	Mannosidase activity	MAN2A2, MAN2B1, KIAA2018, MAN2A1	4
Molecular function	Chaperone binding	TSC1, TP53, TBCE, AHSA2	4
Molecular function	Beta-galactoside alpha-2,3-sialyltransferase activity	ST3GAL2, ST3GAL1	2
Molecular function	Histone methyltransferase activity	PRMT7, SETD1B, PRDM2, SETD2, CARM1	5
Molecular function	Apolipoprotein A-I binding	LCAT, ABCA1	2
Molecular function	Insulin-like growth factor binding	CRIM1, HTRA1, IGFBP5, IGFBP7	4
Molecular function	Mannosyl-oligosaccharide mannosidase activity	MAN2A2, KIAA2018, MAN2A1	3
Cellular component	Membrane fraction	CYP26B1, IGHG3, CYP2D6, SLC12A3, OLR1, SNTB2, MCTP2, JPH2, TNKS, PITPNM1, TSC1, GGCX, CD24, DGCR2, CYP4F2, ENO3, DEGS1, RHBDL1, ADCY6, TSPAN13, DLGAP3, ABCC3, JPH3, PXMP4, SPHK2, L1CAM, ABCA1, ABCC1, SPTAN1	29
Cellular component	Insoluble fraction	CYP26B1, IGHG3, CYP2D6, SLC12A3, OLR1, SNTB2, MCTP2, TP53, JPH2, TNKS, PITPNM1, TSC1, GGCX, CD24, DGCR2, CYP4F2, ENO3, DEGS1, RHBDL1, ADCY6, TSPAN13, DLGAP3, ABCC3, JPH3, PXMP4,	30

		SPHK2, L1CAM, ABCA1, ABCC1, SPTAN1	
Cellular component	Extrinsic to membrane	ANKFY1, CYP26B1, SPRED1, FARP1, CYP2D6, GNG3, SNTB2, JPH2, GOLGA8B, RNF34, TNKS, PITPNM1, GOLGA1, TSC1, PEX5, CYP4F2, DLGAP3, JPH3, PLEKHA4, GCC1, PCYT1A	21
Cellular component	Lamellipodium	TSC1, ABI2, CTTN, PARVA, PSTPIP1, RAPH1, FGD1	7
Cellular component	Cell fraction	IGHG3, OLR1, MCTP2, TP53, JPH2, MAN2B1, FBLN1, TNKS, PITPNM1, ENO3, ADCY6, TSPAN13, CTTN, PXMP4, JPH3, ABCC1, SPTAN1, CYP26B1, CYP2D6, SLC12A3, SNTB2, TSC1, GGCX, CD24, DGCR2, CYP4F2, DEGS1, RHBDL1, DLGAP3, ABCC3, SPHK2, L1CAM, ABCA1, PCYT1A, THOP1, MAPK12	36
Cellular component	Laminin-11 complex	LAMB2, LAMA5	2
Cellular component	Organelle lumen	ZNF207, NCOR2, PRKAB2, TAF1C, TP53, SUDS3, RNF34, CCNL2, MDC1, SIN3A, MED4, CCNE1, HOXA10, THBS1, SARS2, POLR3D, DAZAP1, DMBX1, SPTBN1, LMO4, CBR4, NOC2L, CHD4, IGF2BP1, MAPK7, STAT3, OGG1, SIRT1, CPS1, DMAP1, SETD1B, SLC27A2, SMARCAD1, S100A4, CES1, MKI67, BRD1, TSEN54, ZMYM3, NUMA1, ACO2, TRRAP, DGCR8, TBX3, OGT, PCGF2, MRPS28, GNL3L, FOXF1, SMARCA5, MRPL23, FUBP1	52
Cellular component	Intracellular organelle lumen	ZNF207, NCOR2, PRKAB2, TAF1C, TP53, SUDS3, RNF34, CCNL2, MDC1, SIN3A, MED4, CCNE1, HOXA10, SARS2, POLR3D, DAZAP1, DMBX1, SPTBN1, LMO4, CBR4, NOC2L, CHD4, IGF2BP1, MAPK7, STAT3, OGG1, SIRT1, CPS1, DMAP1, SETD1B, SLC27A2, SMARCAD1, S100A4, CES1, MKI67, BRD1, TSEN54, ZMYM3, NUMA1, ACO2, TRRAP, DGCR8, TBX3, OGT, PCGF2, MRPS28, GNL3L, FOXF1, SMARCA5, MRPL23, FUBP1	51
Cellular component	Membrane-enclosed lumen	ZNF207, NCOR2, PRKAB2, TAF1C, TP53, SUDS3, RNF34, CCNL2, MDC1, SIN3A, MED4, CCNE1, HOXA10, THBS1, SARS2, POLR3D, DAZAP1, DMBX1, SPTBN1, LMO4, CBR4, TXNIP, NOC2L, CHD4, IGF2BP1, MAPK7, STAT3, OGG1, SIRT1, CPS1, DMAP1, SETD1B, SLC27A2, SMARCAD1, S100A4, CES1, MKI67, BRD1, TSEN54,	53

		ZMYM3, NUMA1, ACO2, TRRAP, DGCR8, TBX3, OGT, PCGF2, MRPS28, GNL3L, FOXF1, SMARCA5, MRPL23, FUBP1	
Cellular component	Nuclear lumen	ZNF207, NCOR2, PRKAB2, TAF1C, TP53, SUDS3, RNF34, CCNL2, MDC1, SIN3A, MED4, CCNE1, HOXA10, POLR3D, DAZAP1, DMBX1, SPTBN1, LMO4, NOC2L, CHD4, IGF2BP1, MAPK7, STAT3, OGG1, SIRT1, DMAP1, SETD1B, SMARCAD1, S100A4, MKI67, BRD1, TSEN54, ZMYM3, NUMA1, TRRAP, DGCR8, TBX3, OGT, PCGF2, GNL3L, FOXF1, SMARCA5, FUBP1	43
