# Supplementary material for: Cancer-initiating cells derived from established cervical cell lines exhibit stem-cell markers and increased radioresistance
Source: BMC Cancer. 2012 Jan 28;12:48. doi: 10.1186/1471-2407-12-48 (PMC3299592; doi:10.1186/1471-2407-12-48)

Supplementary Figure 2. Vein diagram for common genes between HeLa and SiHa spheroid cells compared with monolayer cells, whose expression was found up- and down-regulated by a factor of at least 1.5-fold

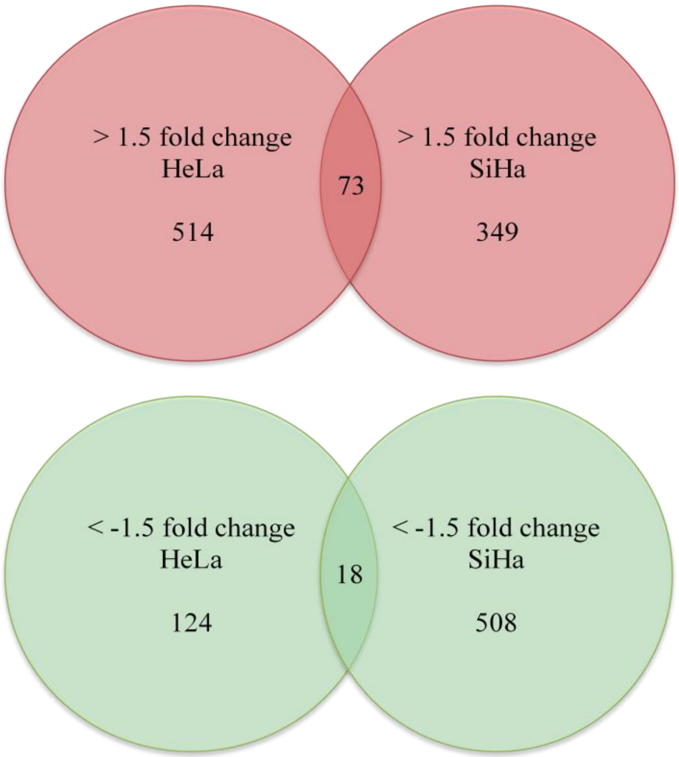

Supplement: Additional file 8 — Figure S2- Genes. Vein diagram for common genes between HeLa and SiHa spheroid cells compared with monolayer cells, whose expression was found up- and down-regulated by a factor of at least 1.5-fold. [file 1471-2407-12-48-S8.PDF]
